# Combining Chemical Information From Grass Pollen in Multimodal Characterization

**DOI:** 10.3389/fpls.2019.01788

**Published:** 2020-01-31

**Authors:** Sabrina Diehn, Boris Zimmermann, Valeria Tafintseva, Stephan Seifert, Murat Bağcıoğlu, Mikael Ohlson, Steffen Weidner, Siri Fjellheim, Achim Kohler, Janina Kneipp

**Affiliations:** ^1^ Department of Chemistry, Humboldt-Universität zu Berlin, Berlin, Germany; ^2^ BAM Federal Institute for Materials Research and Testing, Berlin, Germany; ^3^ Faculty of Science and Technology, Norwegian University of Life Sciences, Ås, Norway; ^4^ Faculty of Environmental Sciences and Natural Resource Management, Norwegian University of Life Sciences, Ås, Norway; ^5^ Faculty of Biosciences, Norwegian University of Life Sciences, Ås, Norway; ^6^ Nofima AS, Ås, Norway

**Keywords:** pollen, consensus principal component analysis, ANOVA simultaneous component analysis, Fourier-transform infrared spectroscopy, matrix assisted laser desorption/ionization mass spectrometry, surface-enhanced Raman scattering, Raman spectroscopy, *Poa alpina*

## Abstract

The analysis of pollen chemical composition is important to many fields, including agriculture, plant physiology, ecology, allergology, and climate studies. Here, the potential of a combination of different spectroscopic and spectrometric methods regarding the characterization of small biochemical differences between pollen samples was evaluated using multivariate statistical approaches. Pollen samples, collected from three populations of the grass *Poa alpina,* were analyzed using Fourier-transform infrared (FTIR) spectroscopy, Raman spectroscopy, surface enhanced Raman scattering (SERS), and matrix assisted laser desorption/ionization mass spectrometry (MALDI-TOF MS). The variation in the sample set can be described in a hierarchical framework comprising three populations of the same grass species and four different growth conditions of the parent plants for each of the populations. Therefore, the data set can work here as a model system to evaluate the classification and characterization ability of the different spectroscopic and spectrometric methods. ANOVA Simultaneous Component Analysis (ASCA) was applied to achieve a separation of different sources of variance in the complex sample set. Since the chosen methods and sample preparations probe different parts and/or molecular constituents of the pollen grains, complementary information about the chemical composition of the pollen can be obtained. By using consensus principal component analysis (CPCA), data from the different methods are linked together. This enables an investigation of the underlying global information, since complementary chemical data are combined. The molecular information from four spectroscopies was combined with phenotypical information gathered from the parent plants, thereby helping to potentially link pollen chemistry to other biotic and abiotic parameters.

## Introduction

The analysis of pollen samples is a crucial task that is necessary in several fields, including agriculture, plant physiology, ecology, allergology, and climate studies. Therefore, significant efforts have been undertaken to utilize analytical techniques that give insight into pollen chemical composition, to achieve a characterization that is more detailed than the morphological typing by light microscopy.

Vibrational spectroscopic methods, such as FTIR (Pappas et al., 2003; Gottardini et al., 2007; Dell'Anna et al., 2009; Julier et al., 2016; Depciuch et al., 2018; Jardine et al., 2019), Raman scattering (Ivleva et al., 2005; Schulte et al., 2008), and surface enhanced Raman scattering (SERS) (Sengupta et al., 2005; Seifert et al., 2016), as well as mass spectrometric methods (Krause et al., 2012; Lauer et al., 2018) can be applied to classify pollen according to taxonomic relationships based on molecular composition. Pollen spectra can also indicate changes in chemical composition according to genetic background and environmental influences (Zimmermann and Kohler, 2014; Zimmermann et al., 2017; Diehn et al., 2018). A vibrational or mass spectrum carries fingerprint-like information from all biomolecular species in the pollen samples that are probed with the respective spectroscopy, albeit with different selectivity and sensitivity (Bagcioglu et al., 2015; Diehn et al., 2018). For example, FTIR spectra of pollen reveal different biochemical composition for different plant species (Pappas et al., 2003; Gottardini et al., 2007; Dell'Anna et al., 2009; Zimmermann, 2010; Julier et al., 2016) and within a specific species (Zimmermann et al., 2017), mainly based on vibrations of protein and lipid molecules contained in the pollen grains. Raman microspectroscopy, since based on different selection rules, can give molecular and structural information complementary to infrared spectroscopy. Moreover, due to the different geometry in Raman micospectroscopic experiments and the penetration depth of the light used to excite the Raman scattering, different parts of the pollen grains are probed. For example, Raman spectra show many contributions by stored starch and lipid bodies and by the sporopollenin polymer that comprises the pollen exine (Schulte et al., 2008). This biopolymer consists of coniferyl aldehyde and ferulic acid blocks (Rozema et al., 2001; Blokker et al., 2006; Li et al., 2019) and provides high stability and protection to the gametes. Surface-enhanced Raman scattering (SERS), in turn, gives very strong signals from pollen constituents that must interact with metal nanoparticles, i.e., the SERS substrate, and although it enables the investigation of less abundant molecular species, it has a high selectivity for specific classes of molecules. This can be the water-soluble pollen fraction, extracted in a facile way (Seifert et al., 2016) or the sporopollenin polymer after embedding the SERS nanoparticle substrate inside the nanoscopic cavities of the pollen shell (Joseph et al., 2011).

In contrast to vibrational spectra, the molecular basis of matrix-assisted laser desorption/ionization time-of-flight mass spectrometry (MALDI-TOF MS) mass spectra from the complex pollen samples is still much less understood (Krause et al., 2012), but it was successfully shown to serve as fingerprint-like data for the classification and identification of pollen species as well (Krause et al., 2012; Lauer et al., 2018), even at the sub-species level (Diehn et al., 2018). MALDI-TOF-MS delivers chemical identifiers that are complementary to those of FTIR spectroscopy when applied to the same set of pollen samples (Zimmermann et al., 2017; Diehn et al., 2018).

Biospectroscopic data are usually evaluated using multivariate methods, including principal component analysis (PCA) (Lasch and Naumann, 1998; Ellis and Goodacre, 2006; Qian et al., 2008). It factorizes the data matrix that contains all spectra to one score value for each spectrum and one loading vector for all spectra. The weighting of the data based on variance in a PCA enables easier identification of differences in a spectral data set and helps identification of latent structures (Pearson, 1901; Hotelling, 1933; Bro and Smilde, 2014). The outcome of a PCA can be explored easily by scores plots and interpretation of spectral features in corresponding loadings.

Since each of the four analytical approaches provides unique information about one particular fraction of the complex pollen chemistry, a combination of the data in one extensive analysis would be very promising to improve pollen characterization and classification. In particular, the combination of different chemical data is expected to reveal chemical aspects of plant/pollen phenotype in a more sensitive and more comprehensive fashion, enabling more insight into, e.g., the adaptation of plants to environmental conditions. Recent studies show the great potential of applying consensus principal component analysis (CPCA) (Wold et al., 1987; Westerhuis et al., 1998) as a multi-block method to the data from different analytical techniques. The combination of very different data blocks can be used in the investigation of biological samples (Perisic et al., 2013), including pollen (Bagcioglu et al., 2015). CPCA is an extension of the PCA concept and aims for the maximization of common variation patterns in the different data blocks. In CPCA, the data blocks are deflated with respect to the variation that is expressed in the so-called global scores. A difference between PCA on every single block and a CPCA analysis is that the same variation appears in the same components in every data block. Thereby, we can compare results directly between the different types of spectroscopic information. A potential co-variation in the blocks is specified in the explained variance of the respective block scores. Furthermore, a correlation loadings plot can be generated as result of a CPCA. CPCA not only joins the information from different data blocks in one analysis, it also enables the evaluation of interactions between the different blocks (Hassani et al., 2010; Hassani et al., 2013).

**Figure 1:**
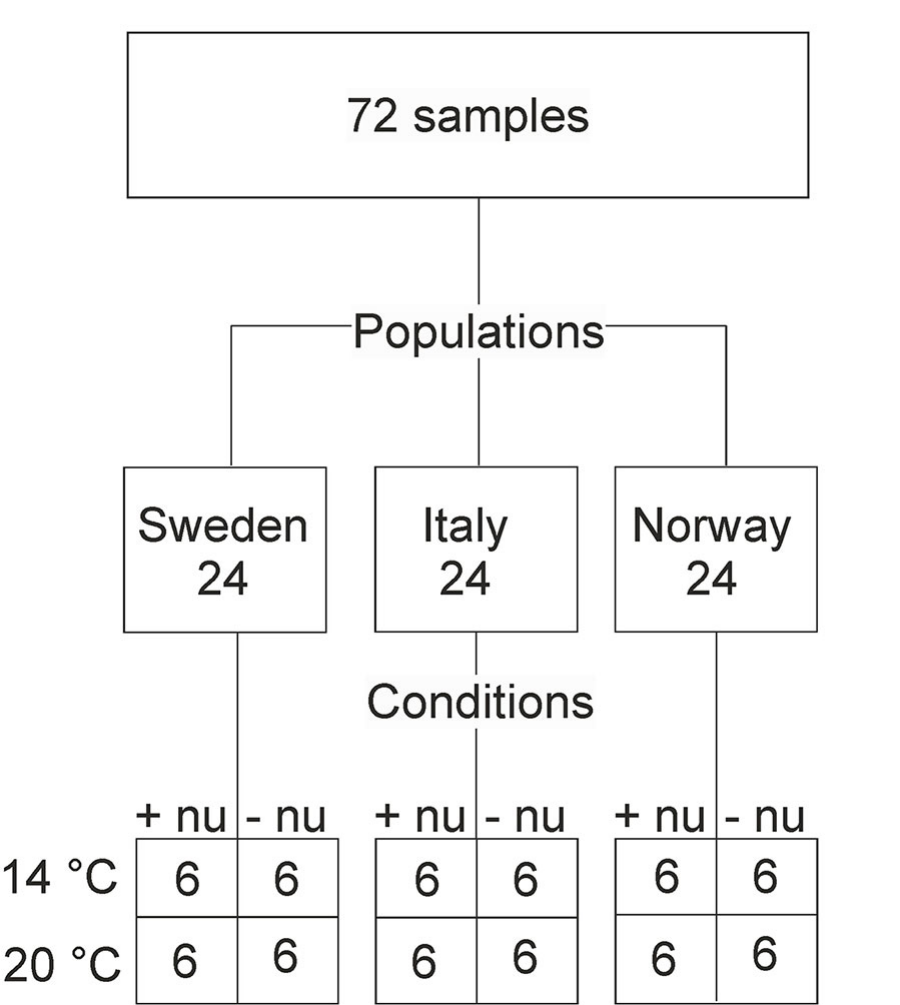
Schematic presentation of the numbers of samples (corresponding to the amount of analyzed spectra) for populations and growth conditions. This results in three (Sweden, Italy, Norway) and four (4°C and additional nutrients, 14°C without additional nutrients, 20°C and additional nutrients, 20°C and without additional nutrients) group variables for the two design factors “population” and “(growth) conditions”, respectively. Abbreviations: +nu, additional nutrients, −nu, no additional nutrients.

Here, we apply CPCA to the pollen data of the four complementary methods FTIR spectroscopy, Raman microspectroscopy, SERS, and MALDI-TOF MS and compare the results to those of PCA of each of the single data blocks. The data are measured from pollen samples obtained in a large-scale greenhouse experiment that was aiming for a diverse range of investigations connecting to pollen research (Zimmermann et al., 2017; Diehn et al., 2018), making also other phenotypic data on the parent plants available to be included in the analysis. The sample set discussed comprises pollen from one grass species, *Poa alpina*. The parent plants originate from three different populations, within which four different growth conditions were applied to individuals of identical genetic constitution (**Figure 1**). The design of this experiment generates two initially separate questions. The first is regarding the different chemical composition of pollen from different populations in the same species. The second question relates to the differences in pollen composition as a result of different growth conditions of genetically identical plants within one population. Therefore, the results of both CPCA and the PCA are compared for the different spectroscopic methods and separately for the different design factors, that is, population and growth condition. One of the aims is to assess the sensitivity of the multimodal characterization towards an influence of population and environmental conditions, respectively, on pollen chemistry, regardless of the hierarchical structure of the variation introduced in the specific sample set. To address this, we have used an ANOVA Simultaneous Component Analysis (ASCA) in order to investigate the possibility of separating between different sources of variation in the complex sample set.

## Experimental

### Pollen Samples

In a greenhouse experiment, plants of the grass species *Poa alpina* were grown under different environmental conditions using seeds acquired from the Nordic Gene Bank. The seeds belonged to three different populations of origin, Sweden, Italy, and Norway, that were chosen to cover geographic and climatic variation (**Figure 1**). Details of the growth experiment can be found in Zimmermann et al. (2017). Briefly, for each population, six individuals were grown from seeds in the spring, and, after the summer, each individual was divided into four clones. The plants were subsequently vernalized for 12 weeks at 4°C with a day length of 8 h. After vernalization, the plants were grown under long day conditions (20 h), and the respective clones of the individuals were subjected to four different environmental conditions: at 14°C and additional nutrients in the irrigation water (+nu), at 14°C without additional nutrients in the irrigation water (-nu), at 20°C +nu, and at 20°C –nu, respectively. Pollen samples were collected from the pollinating plants. Thereby, the sample set contained 24 different pollen samples for each of the three populations, and the whole sample set consisted of 72 pollen samples (**Figure 1**). The pollen grains were stored at -20°C after collection until further preparation.

### FTIR Spectroscopy

Bulk samples of pollen were prepared as homogenous suspensions and measured by using high-throughput FTIR accessory. Approximately 1 mg of a pollen sample was transferred into 1.5 ml microcentrifuge tube containing 500 μl of distilled water. The sample was sonicated in ice bath, by a 2 mm probe coupled to a Q55 Sonicator ultrasonic processor (QSonica, LLC, USA) under 100% power. The sonication period was 2 min in total, with 30 s intermission after the first minute of sonication to minimize the increase in temperature. Following the sonication, the sample suspension was centrifuged with 13,000 rpm for 10 min, and the suspension was concentrated by removing 400 μl of supernatant. Of the remaining suspension, three aliquots (technical replicates), each containing 8 μl, were transferred onto an IR-transparent silicon 384-well microtiter plate (Bruker Optik GmbH, Germany). The microtiter plate was dried at room temperature for 1 h to create adequate films for FTIR measurements.

FTIR measurements were obtained using a HTS-XT extension unit coupled to a TENSOR 27 spectrometer (both Bruker Optik GmbH, Germany). The system is equipped with a globar mid-IR source and a DTGS detector. The spectra were recorded in transmission mode, with a spectral resolution of 4 cm^−1^ and digital spacing of 0.964 cm^−1^. Background (reference) spectra of an empty well on a microtiter plate were recorded before each sample well measurement. The spectra were measured in the 4,000–500 cm^−1^ spectral range, with 32 scans for both background and sample spectra, and using an aperture of 5.0 mm. Data acquisition and instrument control were carried out using the OPUS/LAB software (Bruker Optik GmbH, Germany). Spectra were pre-processed, first by taking the second derivative employing the Savitzky–Golay algorithm (Savitzky and Golay, 1964) with a polynomial of degree two and a window size of 7 points, and second by using extended multiplicative signal correction (EMSC) with linear and quadratic components (Martens and Stark, 1991) (Savitzky and Golay, 1964; Zimmermann and Kohler, 2013). The spectral range from 800–1,800 cm^-1^ was used for multivariate analysis. An average spectrum was calculated from the spectra of the three technical replicates (aliquots) per sample, resulting in a set of 72 average spectra that were used for further analysis.

### Raman Microspectroscopy

Single pollen grains of each pollen sample were measured using a Raman microspectrometer (Horiba, Bensheim, Germany) with a 50x microscope objective (Olympus, Hamburg, Germany) and a diode laser operating at a wavelength of 785 nm and an intensity of 7 · 10^5^ W/cm^2^. For each sample, ten spectra from ten different single pollen grains were collected, using an accumulation time of 10 s per spectrum. In total, 720 individual spectra were obtained. Spectral resolution was 1.3-1.6 cm^-1^, considering the full spectral range. For frequency calibration, six bands in the spectrum of 4-acetaminophenol (1648.4, 1323.9, 1168.5, 857.9, 651.6, 390.9 cm^-1^) were used. After spike removal, the raw spectra were interpolated in the range from 400 to 1,750 cm^-1^ to achieve an equal distribution of data points across the whole spectral range. A distance of 1.45 cm^-1^, corresponding to the average spectral resolution in the experiment was chosen as distance between variables. Subsequently, a baseline for each spectrum was estimated by asymmetric least square smoothing (Eilers, 2003) and subtracted from the respective spectrum, followed by vector normalization of the baseline corrected spectrum. An average spectrum was calculated for each sample from the 10 respective spectra, resulting in a set of 72 average spectra that were used for further analysis.

### Surface-Enhanced Raman Scattering (SERS)

In the SERS experiments, the water-soluble components of the pollen grains were extracted and mixed with an aqueous solution of citrate-stabilized gold nanoparticles as described previously in reference (Seifert et al., 2016). For this purpose, 100 µl Millipore water were added to 0.2 mg of the pollen sample. After 5 min, the samples were centrifuged and the supernatant was pipetted off. 2 µl of this aqueous pollen extract were mixed with 20 µl citrate-stabilized gold nanoparticles obtained based on the protocol described in ref. (Lee and Meisel, 1982) and 2 µl of a 0.1 M sodium chloride solution were added. Subsequently, 20 µl of this mixture were transferred to a calcium fluoride slide for the SERS measurement. The SERS experiments were performed on a Raman microscope (Horiba, Bensheim, Germany) in the focal volume of a 60x water immersion objective (Olympus, Hamburg) with a laser operating at a wavelength of 785 nm and an intensity of 2.9 · 10^5^ W/cm^2^. Two extracts for each sample (technical replicates) were prepared and analyzed. For each extract, 1,000 spectra with an accumulation time of 1 s per spectrum were collected. This procedure yielded SERS data sets containing 144,000 individual spectra in total (2,000 spectra per pollen sample). The spectra were frequency calibrated using a spectrum of 4-acetamidophenol. Further pre-processing included spike removal, interpolation, baseline correction, and vector normalization as described in the previous section. The 2,000 spectra for each sample (obtained from different extracts) were averaged so that in total 72 average SERS spectra were analyzed.

### MALDI-TOF MS

For the MALDI-TOF MS experiments, each pollen sample was deposited on a MALDI stainless steel target. 1 µl of formic acid (90%) was added, and after drying at room temperature, 1 µl of matrix solution (10 mg of α-cyano-4-hydroxycinnamic acid in 1 ml 1:1 acetonitrile/water and 0.1% trifluoroacetic acid) was applied. (Seifert et al., 2015) MALDI-spectra were obtained in the mass range from m/z 1,000 to 15,000 using an Autoflex III MALDI-TOF mass spectrometer (Bruker Daltonik, Bremen Germany) equipped with a 355 nm Smartbeam laser (200 Hz) and operating in positive linear mode at an acceleration voltage of 19.13 kV. Two technical replicates for each sample were prepared, resulting in 144 spectra in total. To obtain equal distances between the variables, the spectra were interpolated with a distance of m/z 2 between data points in the mass range from m/z 5,000 to 9,000, and a 6-degree polynomial baseline correction was applied before the spectra were vector-normalized. The two technical replicates were averaged to yield one spectrum for each sample in order to obtain the same amount of spectra as in each of the other data blocks.

### Morphological and Dry Weight Measurements of Parent Plants

During the pollination stage, the height of the flowering shoots of the parent plants was determined, using the average value for three highest flowering shoots per individual plant. Furthermore, the number of flowering shoots for each individual was determined. Plant dry mass was determined at the end of seed production life stage by cutting the parent plants at ground level and drying them at 60°C for 24 h.

### Chlorophyll Content of Parent Plants

Chlorophyll a and b concentrations were measured by ultraviolet-visible absorption measurements (Solhaug, 1991) Leaf samples from each individual were collected during the pollination life stage. Approximately 4–8 mg of a sample were transferred to microcentrifuge tubes containing 1.5 ml N, N-dimethylformamide, and kept at +4°C for 24 h. The extracts were measured on a UV-Vis spectrophotometer (Shimadzu 1800, Japan) using cuvettes with 1 cm path length. Chlorophyll a and b concentrations were calculated by using absorbance values at 647 nm and 664 nm according to the equations by Porra et al. (Porra et al., 1989). The chlorophyll content, morphological and dry weight data were combined in a separate, fifth data block, termed here additional plant data. The data were normalized based on data dispersion (autoscaling) before further analysis.

### Data Analysis

All pre-processing steps of the spectra and all analyses were performed using the statistics and machine learning toolbox in Matlab R2016a (The Mathworks, Inc., Natick, MA, USA). First, the individual data sets were analyzed by principal component analysis (PCA) as described in previous work (Seifert et al., 2015; Seifert et al., 2016; Joester et al., 2017; Zimmermann et al., 2017; Diehn et al., 2018). Consensus principal component analysis (CPCA) was used to combine the five different kinds of data (FTIR, Raman, SERS, and MALDI-TOF MS spectra, as well as a block with the additional data obtained from the parent plants), each one pre-processed as described before. CPCA enables the analysis of the sample variances within the data blocks and between different data blocks (Hassani et al., 2010). In order to apply CPCA, all the data sets need to have the same sample dimension, and the order of the samples should be identical for all data sets included in the analysis. Therefore, all technical replicates were averaged, such that 72 spectra for each data block were obtained. Outliers were not removed from the averaged data sets, since each method probes different parts of the samples, and the data acquisition differs greatly. For the common representation of the loadings of all the different kinds of data in one correlation loadings plot as result, thresholds were defined for the respective data types and those positive/negative peaks above/below the respective threshold are represented in the unified plot. For clarity, all other spectral variables are not shown in these plots. Only the variables belonging to the additional plant data are displayed as a whole.

To evaluate the ability to discriminate each of the three groups in the design factor “population” as well as the four individual groups of the design factor “growth condition” in the PCA, Kruskal-Wallis H-test and MANOVA were used. The populations Sweden, Italy and Norway, as well as the conditions 14°C and additional nutrients (14+nu), 14°C without additional nutrients (14-nu), 20°C and additional nutrients (20+nu) and 20°C and without additional nutrients (20-nu) are defined as group variables of the design factors “population” and “growth condition”, respectively (**Figure 1**).

Kruskal-Wallis H-test and MANOVA were also applied to the groups of score values after CPCA on the global and block scores, respectively. As result, the two tests give one p-value and one d-value for each PCA or CPCA. The Kruskal-Wallis H-test as a non-parametric statistical test was chosen after assessment of the data sets regarding their normal distribution. As known from previous work, the data obtained from SERS experiments do often not show normal distribution (Seifert et al., 2016). Here, the test is used to prove the null hypothesis that the distribution of the data within each respective group, that is, three groups for the three different populations in the whole data set, and four groups for the four different growth conditions within each of the populations, is equal. A p-value below 0.05 indicates a significant difference in this distribution for at least one of the groups. The Kruskal-Wallis H-test was applied to the score values of each PCA and CPCA that was conducted using the *kruskalwallis* function in Matlab. Each of the first ten components was investigated, and the p-value was reported using always the first PC. However, in case of a high p-value when using the first PC, the lowest p-value with any of the other first ten PCs is discussed (see **Tables 1** and **2**). The distribution of the score values of the first PC is also visualized using box plots.

**Table 1:** Results of the PCA (p- and d-values) for the discrimination of pollen samples from all populations and from the individual populations grown under different environmental conditions.

Method	Population	p-values for the separation of the pollen samples based on environmental conditions	d-values for grouping based on environmental conditions (max. 3)
FTIR	Sweden	<0.01	3
	Italy	0.035	3
	Norway	0.072 (0.011, PC4)	2
	All	0.64 (<0.01, PC6)	3
Raman	Sweden	0.51 (<0.01, PC4)	1
	Italy	<0.01	3
	Norway	0.36 (<0.01, PC3)	2
	All	<0.01	2
SERS	Sweden	0.73 (<0.01, PC2)	2
	Italy	0.37 (<0.01, PC4)	2
	Norway	0.64 *	0
	All	0.78 (0.014, PC7)	0
MALDI	Sweden	0.62 (0.046, PC3)	2
	Italy	0.012	1
	Norway	0.018	1
	All	0.98 (<0.01, PC5)	1
Additional plant data	Sweden	<0.01	1
	Italy	<0.01	2
	Norway	<0.01	2
	All	<0.01	2

*no p-value below 0.05 can be found for the first 10 PCs. The p-values are obtained for the score values of PC1. In case of p-values above 0.05 in PC1, the lowest p-value with any of the other first ten PCs and respective PC are shown in parentheses. For the calculation of d-values, the score values of the first 10 PCs were used.

**Table 2:** Results of the CPCA (p- and d-values) for the discrimination of pollen samples from all populations and from the individual populations grown under different environmental conditions.

Method	Population	p-values for the separation of the pollen samples based on environmental conditions	d-values for grouping based on environmental conditions (max. 3)
Global	Sweden	0.036	2
	Italy	<0.01	3
	Norway	<0.01	2
	All	0.95 (<0.01, CPC3)	3
FTIR	Sweden	0.032	2
	Italy	0.013	3
	Norway	0.084 (0.021, CPC4)	3
	All	0.70 (0.011, CPC3)	3
Raman	Sweden	0.47 (0.013, CPC6)	2
	Italy	<0.01	3
	Norway	0.19 (<0.01, CPC5)	3
	All	0.64 (<0.01, CPC3)	3
SERS	Sweden	0.093 (0.032, CPC5)	2
	Italy	0.089 (<0.01, CPC3)	3
	Norway	0.40 *	2
	All	0.98 (<0.01, CPC3)	3
MALDI	Sweden	0.60 (0.027, CPC5)	2
	Italy	<0.01	3
	Norway	<0.01	3
	All	0.98 (<0.01, CPC3)	2
Additional plant data	Sweden	<0.01	2
	Italy	<0.01	3
	Norway	<0.01	2
	All	<0.01	3

*no p-value below 0.05 can be found for the first 10 PCs. The p-values are obtained for the score values of PC 1. In case of p-values above 0.05 in PC 1, the lowest p-value with any of the other first ten PCs and respective PC are shown in parentheses. For the calculation of d-values, the score values of the first ten PCs were used.

MANOVA, comparing the multivariate means for a specific dimensionality, was executed using the *manova1* function in Matlab. The first ten PCs (covering at least ~90% of the variance, with over ~96% of the variance in the FTIR and Raman data sets) were used for MANOVA, since a balance had to be found between the requirement to have as much variance as possible covered, an equal treatment of all data sets, and a reasonable time for computation. MANOVA was used to estimate the non-random variation of the group mean of each population and each growth condition, respectively. In this case, the dimensionality was either three, corresponding to the three different populations, or four, due to formation of four groups corresponding to the four different growth conditions. If the group means were equal, that is, when no discrimination was found, the d-value would be 0. A d-value of 1 would indicate that the group means show a linear dependence on each other, so that two groups are separated. For the data sets here, the d-value could reach two in the case of the three different populations and three in the case of the four different growth conditions.

To calculate the variation in the data induced by the different design factors, such as population, nutrients, temperature, as well as their interaction, we used an approach underlying ANOVA-PCA and ASCA (Harrington et al., 2005; Smilde et al., 2005), which are widely used for this purpose (Jansen et al., 2005; de Haan et al., 2007). In both of these methods an ANOVA model is established. It represents the original data as a sum of matrices, each of which corresponds to one design factor. Each of these matrices consists of the means of the spectra that correspond to different levels of the factor. As an example, if one design factor has two levels, its respective matrix will have repeated means of the two levels in the corresponding rows.

In the ANOVA model used in this study, the design factor “temperature” has two levels (14°C and 20°C), the design factor “nutrients” consists of two levels (+nu and -nu), the design factor “interaction” is the interaction of ‘temperature' and ‘nutrients' and has four levels, the design factor “population” has three levels (Italy, Norway, and Sweden), the factor ‘individual' has 72 levels that correspond to each individual plant in the set of samples. The residual variance is summarized in the factor “residuals”.

ASCA uses the ANOVA model to study the effects of the design factors on the variation in the data and runs PCA on each of the matrices to interpret this variation. In ANOVA-PCA the ANOVA model is analyzed further by PCA to find if the differences in the levels for each factor are significant. In this study, we use the ANOVA model purely to calculate each design factor contribution into variation in the data. Since it was of interest to learn about the variation contributions in each block of data representing each measurement (FTIR, MALDI, Raman, SERS and other parental plant data, respectively), the same analysis was done separately on each data block.

## Results and Discussion

### Analysis of the Separate Data Blocks

The well-defined sample set (**Figure 1**) was measured by the four different methods FTIR, Raman, and SERS spectroscopy and MALDI-TOF mass spectrometry, after different pre-processing of the samples according to the requirement of each spectroscopy (see *Experimental* section), leading to the probing of complementary constituents of the pollen. The four different types of spectra were obtained from the 72 pollen samples, constituting four separate data blocks. Furthermore, phenotypic information from the respective parent plants was combined in a fifth data block.

**Figure 2:**
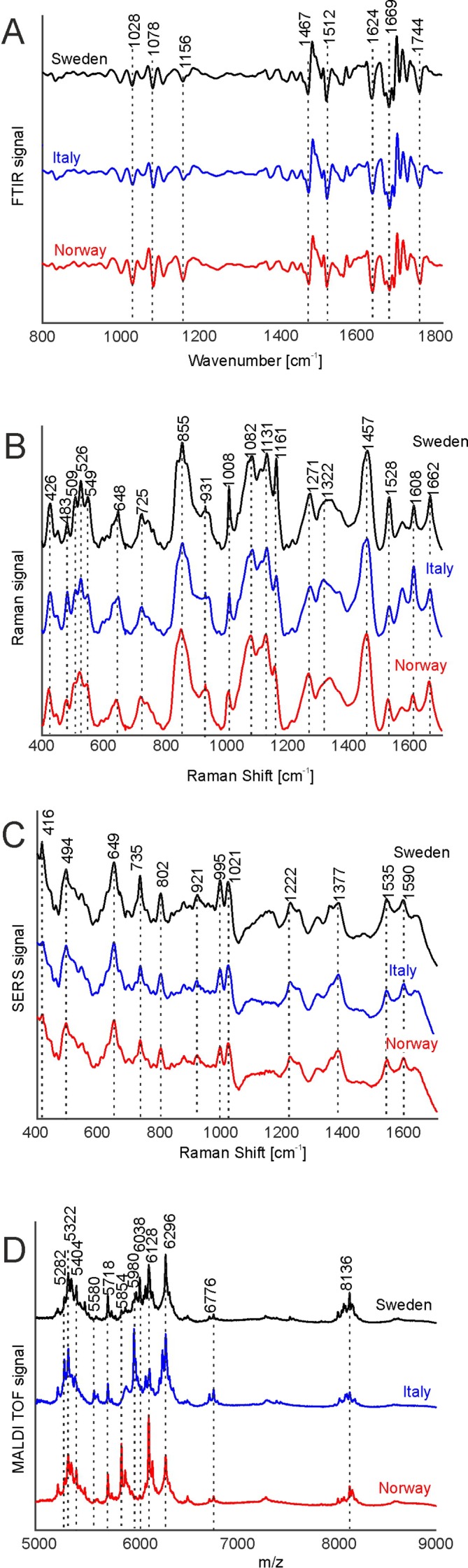
FTIR **(A)**, Raman **(B)**, SERS **(C)**, and MALDI-TOF MS **(D)** spectra of pollen from the populations Sweden (black), Italy (blue), and Norway (red). All spectra were pre-processed according the requirements of the respective spectroscopic method and are averages from the respective population, including samples obtained for all growth conditions. The spectra are stacked for clarity. FTIR, Fourier-transform infrared spectroscopy; Raman, Raman spectroscopy; SERS, surface enhanced Raman scattering; MALDI-TOF MS, matrix assisted laser desorption/ionization mass spectrometry.

**Figure 2** shows the four types of spectra obtained for the three different populations, with averaging information in each population over pollen samples obtained from all parent plants grown at the four different environmental conditions. The signals in the FTIR spectra (**Figure 2A**) can mainly be assigned to proteins, represented, e.g., by the amide I and amide II bands at 1669 and 1540 cm^-1^, respectively, to lipids, exemplified by vibrations at 1156, 1467, and 1744 cm^-1^ and to sporopollenin, e.g., at 835, 1512, and 1624 cm^-1^, in agreement with spectra reported previously (Bagcioglu et al., 2015; Zimmermann et al., 2017).

The average Raman spectra in **Figure 2B** are very similar to each other as well and display similar signals, albeit at slightly varying positions, suggesting small differences in the chemical composition of pollen from different populations. The bands at 1008, 1161, and 1528 cm^-1^ can be assigned to carotenoids (Schulte et al., 2009), while the signals at 526, 549, 725, 855, 1271, 1457, and 1662 cm^-1^ are due to vibrations of proteins (Schulte et al., 2008; Joester et al., 2017). The bands at 483, 1082, and 1322 cm^-1^ are assigned to carbohydrates (Schulte et al., 2008; Pigorsch, 2009) that can occur at high local concentrations in the pollen grains as starch deposits. Due to superposition of several molecular vibrations, some bands in the Raman spectra of pollen can be assigned to other origins as well. As examples, the bands at 1161, 1271, 1313, and 1608 cm^-1^ could also be assigned to the ferulic acid and coumaric acid building blocks in sporopollenin (Blokker et al., 2006; Bagcioglu et al., 2015). Furthermore, the band at 1608 cm^-1^ has also been associated with mitochondrial activity (Huang et al., 2004; Pully and Otto, 2009).

Due to the sample preparation as aqueous extract and the use of aqueous nanoparticle solutions, the SERS experiments probe the water-soluble fraction of the pollen grains. Because of the high variation in the SERS spectra caused by the specifics of the SERS experiment, high numbers of spectra are needed for a reliable statistical analysis. (Seifert et al., 2016) Therefore, 2,000 spectra were measured from each sample, resulting in reproducible average spectra that are based on 24,000 individual spectra per population. They are shown in **Figure 2C**. The average spectra are very similar, and their characteristic bands can mainly be assigned to vibrational modes of nucleobases, e.g., at 494, 649, 735, 802, 921 cm^-1^ (Seifert et al., 2016) and amino acids, at 995, 1021, 1221 cm^-1^ (Kyu et al., 1987; Stewart and Fredericks, 1999), in agreement with the probing of water-soluble biomolecules extracted from the pollen.

MALDI TOF mass spectrometry was utilized to detect large molecules with a mass over 5 k Dalton. **Figure 2D** displays average MALDI-TOF mass spectra showing peaks at m/z 5282, 5322, 5404, 5580, 5718, 5853, 5980, 6128, 6296, 6776, and 8136. The differences in the population averages are obvious and indicate that the pollen samples differ in their composition in each population. From earlier attempts to interpret the bands we infer that they include oligosaccharides (Krause et al., 2012; Seifert et al., 2015; Diehn et al., 2018) and larger peptides.

**Figure 3:**
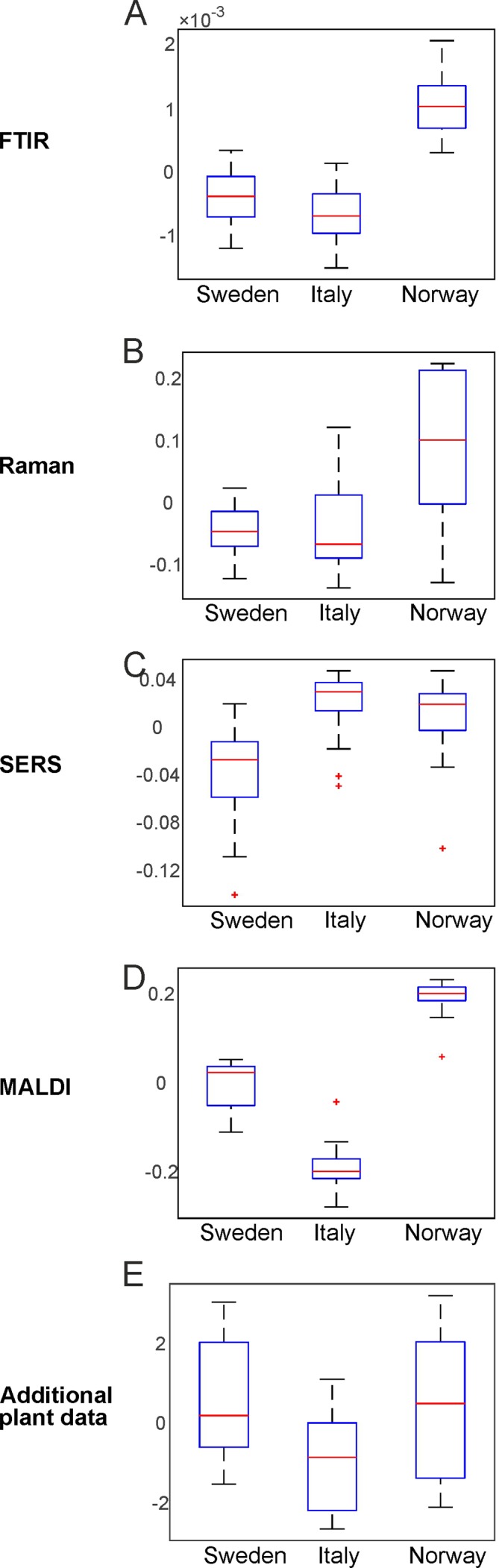
Results of the principal component analysis of FTIR **(A)**, Raman **(B)**, SERS **(C)**, MALDI-TOF MS **(D)**, and additional plant data **(E)**, respectively. Box plots display the variation of the score values of the first PC regarding each population obtained by Kruskal-Wallis H-Test. Red lines indicate the median of the respective distribution, blue boxes represent the interquartile range, the black lines demarcate minimum and maximum values, and extreme values are shown as red markers. FTIR, Fourier-transform infrared spectroscopy; Raman, Raman spectroscopy; SERS, surface enhanced Raman scattering; MALDI-TOF MS, matrix assisted laser desorption/ionization mass spectrometry.

By PCA of the respective type of spectra/data, the pollen samples of the three different populations can be discriminated using each of the individual data blocks. To visualize the distribution of the score values of PC1 for each population, **Figure 3** shows the corresponding box plots with minimum and maximum score values. Outliers are mainly observed for the SERS data (**Figure 3C**) due to high variation in this type of data owing to the specific measurement approach (Seifert et al., 2016). P-values below 0.05 for all five data sets indicate a separation of at least one group for all these data sets.

The box plots of **Figure 3** show that an unequivocal separation of all three populations based on PC1 is only possible when the MALDI-TOF MS scores (**Figure 3D**) are used. The scores of FTIR (**Figure 3A**) and Raman data (**Figure 3B**) for example show very similar distributions for the two populations Sweden and Italy. In order to include more than one principal component when evaluating separation of the three populations by PCA, d-values were determined by MANOVA of the scores of the first ten principal components of each PCA/data block. For all data blocks a d-value of 2 is obtained. This indicates the separation between three groups, corresponding here to the three populations. Therefore, we conclude that a separation of the three different populations is possible with any of the data sets.

The parent plants in each population were grown under four different conditions. Discrimination regarding potential effects of additional nutrients and temperature as design factors on pollen chemistry was studied using each of the five data sets separately as well. This was done for each population individually, as well as for all populations together. **Table 1** summarizes for each data block the PCA results. The p-values were determined using one PC (**Table 1**, left column). In case of a high p-value when using the first PC, the lowest p-value with any of the other first ten PCs is shown in the table. The d-values were determined using the first ten principal components (**Table 1**, right column).

The first section of **Table 1** displays the outcome of the PCAs obtained from the FTIR data sets. The separation based on FTIR spectra receives a p-value below 0.05 and a d-value of 3 for the populations Sweden and Italy, indicating that FTIR data alone enable differentiation of the applied growth conditions for these two populations (compare also the box plots in **Figures S2** and **S3**). The FTIR data set of the population Norway with p-value larger than 0.05 and a d-value of 2 comprises less variance between growth conditions. When all populations are analyzed together, a high p-value for the first PC is obtained, which means that none of the four different growth conditions is separated using the variance explained by the first PC. Nevertheless, using the 1st to 10th PC, the d-value of 3 indicates a possible separation of all four growth conditions by FTIR alone.

Using the Raman data sets, the p and d-values of the PCAs from the data of the populations Sweden and Norway indicate a less sufficient discrimination ability (**Table 1**, second section). Only for the population Italy, a low p-value and a d-value of 3 can be interpreted as a separation of the four groups of the different growth conditions. In addition, the analysis of all populations together leads to a small p-value, showing the separation of at least one group based on Raman spectral information. We attribute the smaller discrimination ability compared to FTIR to this different selectivity of Raman spectroscopy. The high variances according to the growth conditions in the population Italy explained by the first PC are remarkable, and in good agreement with previous studies on phenotypic plasticity in pollen (Zimmermann et al., 2017). The higher the phenotypic plasticity, the more the chemical composition in pollen varies when environmental conditions change. The high phenotypic plasticity of the population Italy has been inferred from MALDI TOF MS and FTIR spectra of the same *Poa alpina* population previously (Zimmermann et al., 2017; Diehn et al., 2018), where a lower inner-group variance regarding different genotypes of the plants was found.

Investigation of the SERS spectra from aqueous pollen extract by PCA results in p-values above 0.05 for each individual population as well as the whole data set (**Table 1**, third section), clearly showing that an analysis of the samples by SERS alone will not be sufficient for the discrimination of pollen from parent plants that were grown under different environmental conditions. Nevertheless, according to the p-values found in PC2 in population Sweden and PC4 in population Italy (p-values in parentheses in **Table 1**), the variances from the effect of the growth conditions can also be detected in the aqueous extract for these two data sets and therefore add complementary information in the multi-block analysis discussed below.

The MALDI TOF MS data from population Sweden lead to a p-value above 0.05, whereas the p-values for the other two populations stay slightly below 0.05 (**Table 1**, fourth section). In contrast, based on the outcome for the whole population, we cannot conclude chemical variance as result of different growth conditions using only one PC. The d-value of 1 (obtained using the first ten PCs), found for the whole data set as well as for population Italy and population Norway, can be interpreted as the formation of two distinct groups of MALDI spectra. This is in agreement with our previous results (Diehn et al., 2018), where we found a high ability to discriminate between pollen from plants growing with additional nutrients and pollen from plants without additional nutrients using MALDI data from the same samples of *Poa*. Since the discrimination takes place in the range m/z 5000-9000, we infer that the detected signals belong to proteins and their derivatives from pollen nutrient storage.

The last section in **Table 1** contains the p- and d-values for the analysis of additional plant phenotype data, namely height and number of flowering shoots, plant dry mass, and chlorophyll content. The p-values for each population and for the data set with all three populations combined are below 0.05, and we conclude that the variances regarding the separation of at least one specific group of scores from the other growth conditions are high. The d-values for the analyses of the data sets, however, are 2 or smaller, indicating that discrimination regarding all four growth conditions is not obtained. This is also be illustrated in the box plots in **Figures S1–S4** (last rows).

**Figure 4:**
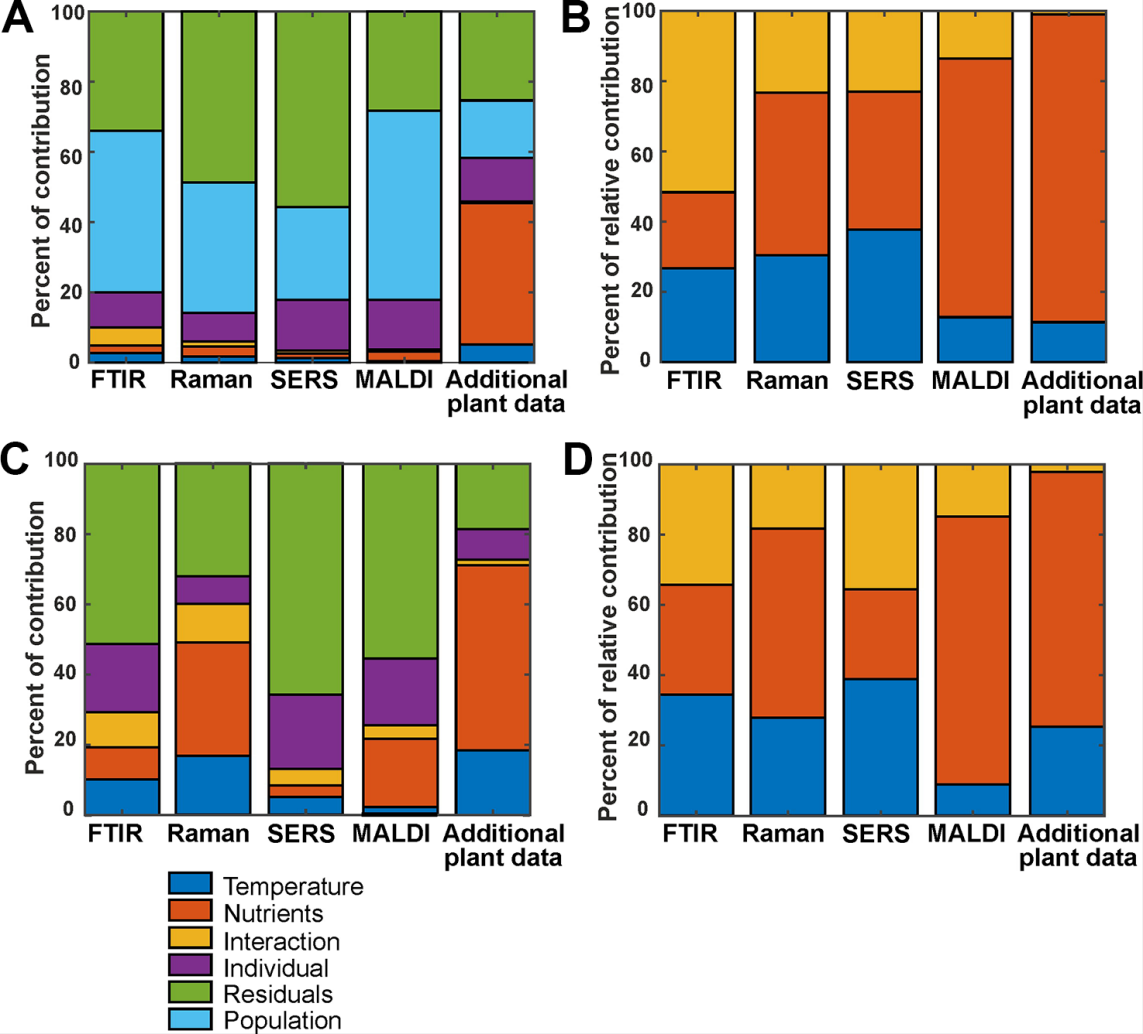
**(A)** Variation contribution of the design factors temperature (blue), nutrients (orange), the interaction of temperature and nutrients (yellow), different individuals (purple), populations (cyan), and residual variance (green) for the 72 spectra from the whole data set. **(B)** Relative contribution of temperature (blue), nutrients (orange), and the interaction of both (yellow) for the 72 spectra from the whole data set **(C)** and variation contribution of the design factors temperature (blue), nutrients (orange), the interaction of temperature and nutrients (yellow), individuals (purple) and the residuals (green) for the 24 spectra from the population Italy. **(D)** Relative contribution of temperature (blue), nutrients (orange), and the interaction of both (yellow) for the 24 spectra from the population Italy. In **(B, D)**, the contribution to the variance by specific population and the residual variance were left out, and the variation of each factor was normalized by the sum of the variations for the three other factors of interest.

The variation contributions of the different design factors, such as population, nutrients, temperature and their interaction as well as the contributions from individual variation, were calculated with an approach underlying ANOVA-PCA and ASCA (Harrington et al., 2005; Jansen et al., 2005; Smilde et al., 2005; de Haan et al., 2007). **Figure 4A** shows the contribution of all possible design factors, that is, each type of variation for the whole data set of 72 spectra for each method. The variation contribution of the populations (**Figure 4A**, cyan bars) is very large in the four spectroscopic/spectrometric data sets, larger than the variation contribution due to the different growth conditions (**Figure 4A**, blue, orange and yellow bars). Interestingly, and in agreement with previous work (Zimmermann et al., 2017), the contributions of variation of the individual samples (**Figure 4A** first column, purple bars) is of similar magnitude as that introduced by changes in growth condition of the parent plant, and in the data sets from SERS and MALDI (**Figure 4A** second and third column, purple bars), this contribution by individual variation is even larger.

Considering the data gathered from the parent plants, the largest variation contribution is the effect of the nutrient addition (**Figure 4A**, rightmost bar, orange coloring), obviously having more consequences for the constitution of the plant itself than for the chemical make-up of the pollen. In addition, differences between phenotypic features of the plants in the different populations are of a similar magnitude as variation due to individual differences. (**Figure 4A**, rightmost bar, cyan coloring). The contribution of the residual variation (**Figure 4A**, green bars) is relatively high for all data sets. In some, such as Raman and SERS (**Figure 4A**, second and third column, respectively), the residual variation contributes the most. We think that this must be due to the type of experiment, which are in these cases much more prone to spectrum-to-spectrum fluctuation. Moreover, the Raman and SERS data sets were collected over a course of several weeks, whereas MALDI and FTIR were high-throughput measurements obtained in one-preparation procedures. So, the big residual variation in SERS and Raman can be explained by the experimental variations.

In **Figure 4B**, relative variation contributions of the growth conditions, namely temperature, nutrients and the interaction of both factors are presented. Variations by these factors are emphasized by omitting population variation, individual variation, and residual variation. To calculate these, a variation of each factor was normalized by the sum of the variations for the three factors of interest. While the variation contribution of both, the temperature and nutrients is high for the three spectroscopic methods FTIR, Raman and SERS, for MALDI the variation of the nutrient factor is higher than the variation contribution of the temperature.

The contribution of the different design factors to the total variation were also analyzed for each population separately (**Figures 4C, D**, **Figure S5**). As an example, **Figures 4C, D** show the outcome of the analysis for the population Italy. For the population Sweden (**Figures S5A, B**), the overall variation contribution of the individuals (**Figure S5A**, purple), is higher compared to the other populations, and contribution of variation due to the growth conditions is rather small.

This type of analysis helps understanding the underlying variation in the data introduced by different design factors and by other unwanted factors. PCA analysis and other multivariate data analysis techniques, if successfully working on the data, ensure that the amount of relevant variation available in the data is sufficient to discriminate between groups. As an example, although the different growth conditions contribute to only 10% of the variation in the FTIR data from all populations (**Figure 4A**, first column), we observed a good discrimination of growth conditions using the first ten PCs, yielding a d-value of 3 (**Table 1**, first section). This shows that the methods are powerful enough to focus on the relevant information in the data, and that the residual variation is not systematic. Regarding the hierarchical nature of the variance, the results of the ASCA approach are in good agreement with the results obtained by PCA. In data sets that show large contributions by different sources of variation, separation in a PCA is not unequivocal (see **Table 1**).

In conclusion, the different analytical methods vary greatly in their potential to discriminate the pollen from the sample set based on population and environmental influences. For FTIR spectroscopy (Zimmermann et al., 2017) and MALDI-MS (Diehn et al., 2018) this has been discussed previously. Due to the different selectivity in MALDI compared to FTIR, there is a superposition by the variation between the different genotypes (that is, individual variation) that impairs the discrimination ability for different growth conditions within one population (Diehn et al., 2018). While both Raman micro-spectroscopy of single pollen grains and SERS enable classification of the pollen samples with respect to the corresponding population, no strong variation is found when these data sets are used to assess separation according to the varied environmental conditions of the parental plants. Nevertheless, the variation due to varied growth conditions is highly dependent on the considered population.

### CPCA for the Classification of Pollen Samples According to Plant Populations

**Figure 5:**
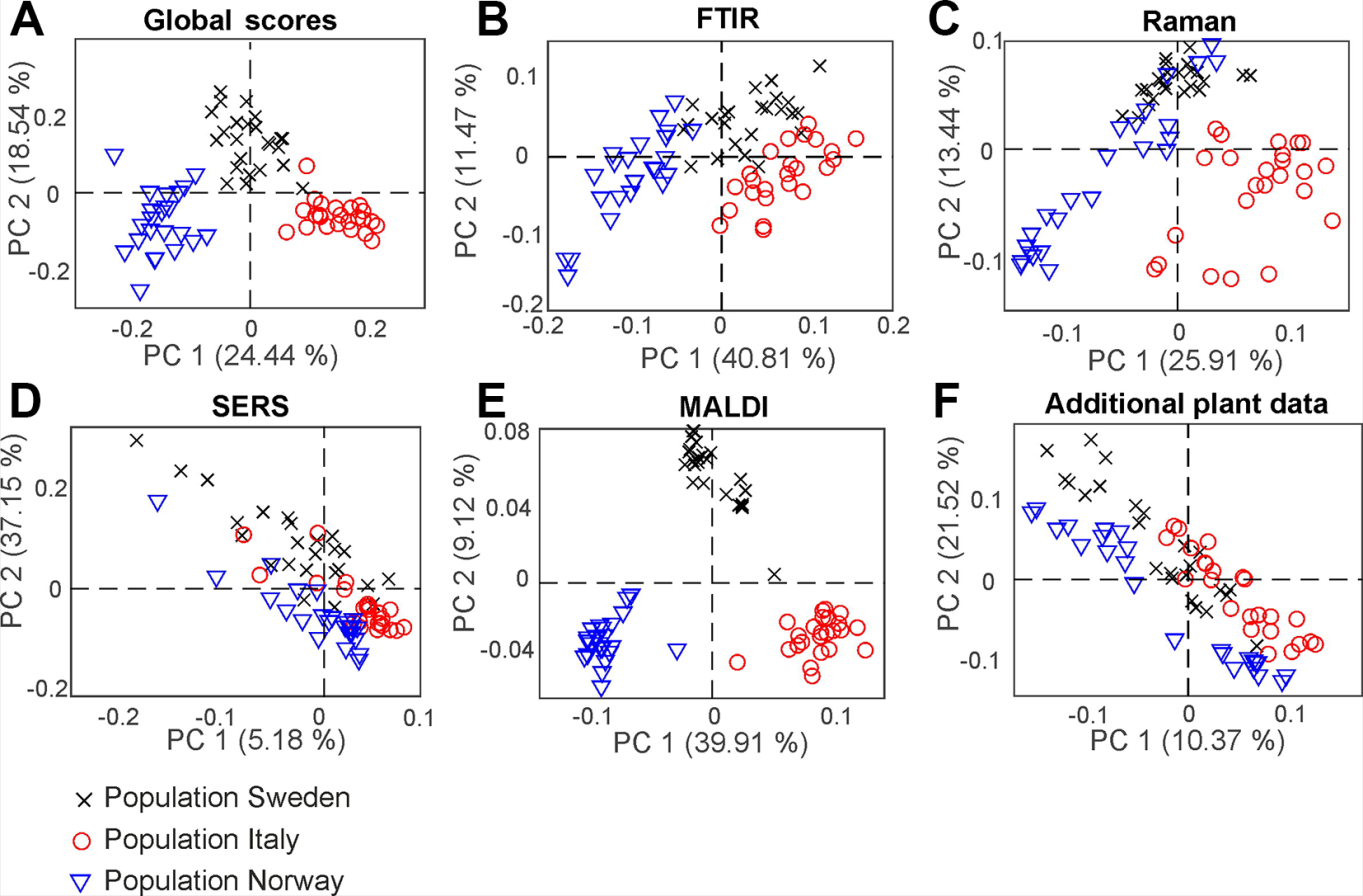
Score values of the CPCA analysis for the classification of samples from the populations Sweden (black crosses), Italy (red circles), and Norway (blue triangles). **(A)** Scores for the global scores **(B–F)** individual data blocks. CPCA, consensus principal component analysis.

With consensus principal component analysis (CPCA), the five individual data blocks can be combined, and the impact of each method on a global analysis can be evaluated. **Figure 5** shows the results of the CPCA for the classification of the different populations of *Poa alpina*, consisting of five block score plots (**Figures 5B–F**) that correspond to the different analyses, and of a global scores plot (**Figure 5A**). The global score values of the first and second PC (**Figure 5A**) show a clear discrimination of the three different populations. In particular, based on the variance represented by CPC1, data from the population Norway and data from the population Italy are separated. As revealed by the block scores plots, the first component is mostly influenced by the FTIR block, comprising 41.7% explained variance (**Figure 5B**) and the MALDI block, explaining 39.62% of the variance (**Figure 5E**). The second PC is influenced in particular by the SERS data, explaining 37.55% of the variance (**Figure 5D**) and the block with the data on the parent plants, explaining 21.84% of the variance (**Figure 5F**). In all of the scores plots, the data sets of the population Sweden have positive score values, while the data sets of the populations Italy and Norway have mostly negative values regarding CPC2 (**Figure 5**), particularly for the Raman (**Figure 5C**), SERS (**Figure 5D**), and MALDI (**Figure 5E**) block. A CPCA containing FTIR, Raman, SERS and MALDI without the additional plant information leads to very similar scores plots, where also all three populations would be discriminated (**Figure S6**).

**Figure 6:**
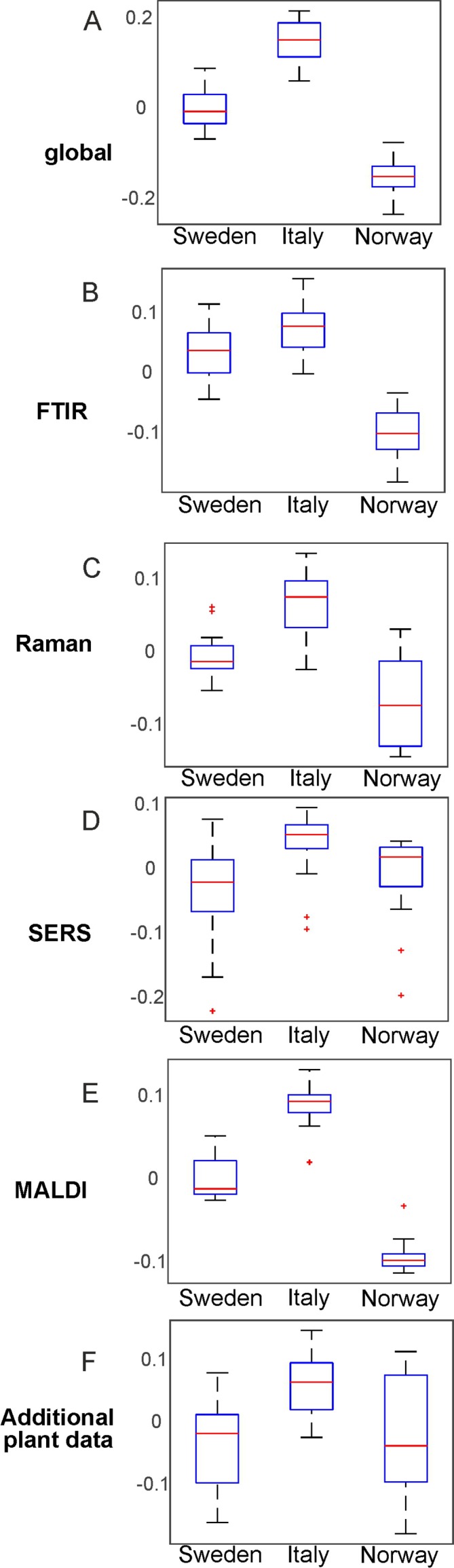
Results of the consensus principal component analysis of the five data sets visualized by box plots of the global scores **(A)** and the blocks scores for FTIR **(B)**, Raman **(C)**, SERS **(D)**, MALDI-TOF MS **(E)**, and additional plant data **(F)**. The box plots display the variation of the score values of the first CPC regarding each population obtained by Kruskal-Wallis H-Test. Red lines indicate the median of the respective distribution, blue boxes represent the interquartile range, the black lines demarcate minimum and maximum values, and extreme values are shown as red markers. FTIR, Fourier-transform infrared spectroscopy; Raman, Raman spectroscopy; SERS, surface enhanced Raman scattering; MALDI-TOF MS, matrix assisted laser desorption/ionization mass spectrometry.

In **Figure 6**, the results of the separation of the respective first CPC are summarized in box plots for each block as well as for the global scores (**Figure 6**). Furthermore, we calculated a d-value of 2 based on the CPCA scores of CPC1 to CPC10 for the global scores as well as for all block scores. The data indicate that separation of the three populations is readily achieved based on the global scores (**Figure 6A**), and that the FTIR (**Figure 6B**) and the MALDI data sets (**Figure 6E**) have the greatest influence on the separation in the global scores.

**Figure 7:**
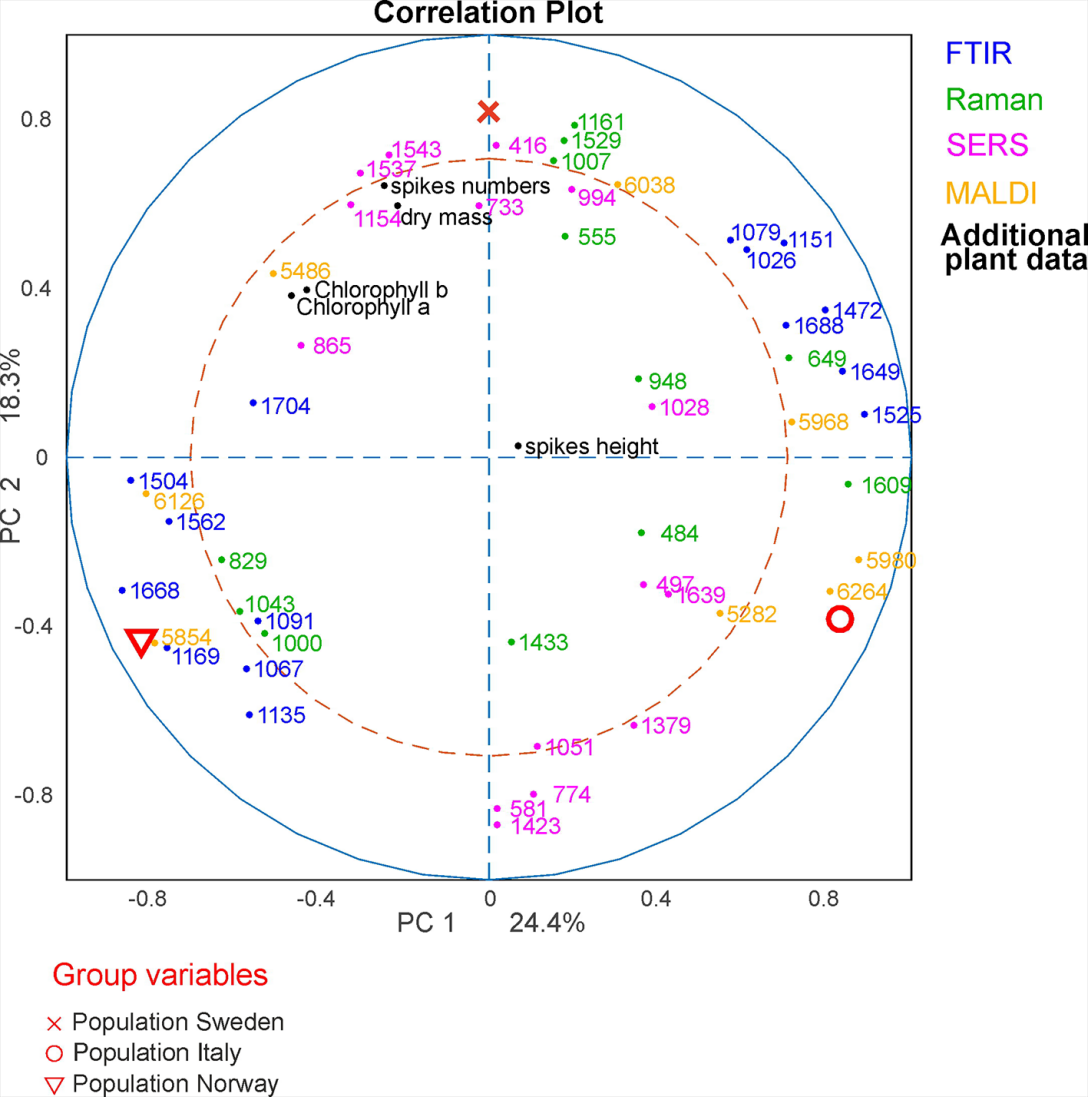
CPCA correlation loadings plot for the 1st and 2nd CPCA component. Displayed are the global scores of the three populations Sweden, (red cross), Italy (red circle), and Norway (red triangle), as well as the loadings of the blocks of FTIR, Raman SERS, MALDI-TOF and additional plant data. For clarity only extrema of the loadings were shown for the spectroscopic/spectrometric data. CPCA, consensus principal component analysis; FTIR, Fourier-transform infrared spectroscopy; Raman, Raman spectroscopy; SERS, surface enhanced Raman scattering; MALDI-TOF MS, matrix assisted laser desorption/ionization mass spectrometry.

In order to analyze which variables of the respective methods cause the separation in the global analysis and to investigate the correlations between them, a correlation loadings plot was generated (**Figure 7**). It shows the correlation between the global scores of the populations Sweden (red cross), Italy (red circle) and Norway (red triangle) and the relevant variables of the different blocks. For the clarity only the extrema of the loadings of the first and second component from the spectroscopic and MALDI blocks are shown, as well as all five variables from the additional plant data. Therefore, there are no variables visible close to the origin of the plot. The different populations are characterized by variables that are located close to the global scores of the populations. The separation of the data from the population Sweden is caused by a high amount of spikes in the respective progenitor plants and their high dry mass. In addition, this population is characterized by Raman bands at 1007, 1161, and 1529 cm^-1^ that can be assigned to carotenoids (Schulte et al., 2009) as well as by bands at 555 cm^-1^ that can be assigned to proteins (Schulte et al., 2008), and a MALDI peak at m/z 6038. The great influence that the SERS data block has on CPC2, separating population Sweden (see **Figure 5C**), reflects in a correlation with SERS signals at 416, 733, 994, 1154 and 1545 cm^-1^ that are particularly important to discriminate the pollen data from the population Sweden (**Figure 7**, magenta markers). In the two other populations, SERS signals at 581, 774, 1051, 1379, and 1424 cm^-1^ are observed. They might be attributed to the water-soluble part of proteins or carbohydrates.

The differentiation between the populations from Norway and Italy is achieved utilizing CPC1. The population from Italy is mainly separated by chemical information contained in the FTIR bands (**Figure 7**, blue markers) at 1026, 1079, 1151, 1472, 1525, 1649, and 1688 cm^-1^, Raman bands (**Figure 7**, green markers) at 484, 649, 948, and 1609 cm^-1^, and MALDI TOF MS peaks (**Figure 7**, yellow markers) at m/z 5282, 5968, 5980, and 6264. The FTIR and Raman bands can be assigned to starch, protein and sporopollenin vibrations (Schulte et al., 2008; Zimmermann, 2010; Bagcioglu et al., 2015). Although an assignment of the MALDI peaks is more challenging, their positive correlation with these bands suggests that some of them are connected to nutrients, in agreement with previous discussions suggesting their assignment to oligosaccharides (Krause et al., 2012; Seifert et al., 2015; Diehn et al., 2018).

The data sets of the population Norway show a positive correlation to the FTIR vibrational bands at 1089, 1166, 1503, 1666, and 1746 cm^-1^ as well as to the MALDI peaks at m/z 5880 and 6296 (**Figure 7**, bottom left section). Variances in Raman bands at 829 and 1043 cm^-1^ are positively correlated to the population Norway. Most of the Raman bands can be assigned as protein vibrations (De Gelder et al., 2007), whereas the FTIR bands could be mainly assigned to carbohydrates (Bagcioglu et al., 2015; Zimmermann et al., 2017).

As illustrated by the band assignments, in addition to a redundancy in information (e.g., in some bands in FTIR and Raman spectra) each data block contains some exclusive molecular information, leading to their complementarity. The different contribution of the five data blocks in the discrimination of the three populations shown in the correlation plot (**Figure 7**) indicates that particular parts of the pollen chemistry are responsible for the differences between populations, and that very different molecular/compositional parameters are responsible in the biochemical variation between two populations. The MALDI-TOF MS data have great influence on the analysis and can be exploited for a precise discrimination of all three populations. This is in accordance with the results of the PCA of the isolated data block above (**Figure 3D**) in this paper and supports previous results that indicate that MALDI-TOF MS and the biochemical fingerprint of glycoproteins and other macromolecules are specific for the pollen of a particular grass population (Diehn et al., 2018).

### CPCA for the Classification of Pollen Samples According to Different Environmental Influences

CPCA was applied as well to discriminate between pollen samples within each population that were collected from progenitor plants grown under four different environmental conditions: 14°C and additional nutrients, 14°C without additional nutrients, 20°C and additional nutrients, 20°C without additional nutrients. **Table 2** shows the resulting p and d-values analyzing the whole data set from all populations and the data from each of the three different populations individually for the global scores (**Table 2**, first section) and all the block scores (second to sixth section, respectively). The p-values for the global scores are below 0.05 for each population, indicating the separation of the different groups in the first CPCA component (**Table 2**, first section). However, considering all three populations together, separation is based on the third CPCA component. MANOVA utilizing the first ten CPCA components shows the highest possible d-value of 3, proving successful classification of all four groups of samples for population Italy, as well as the for the whole sample set of all three populations. The lower d-value for the global scores in the population Sweden and Norway may be explained by a lower phenotypic plasticity of these populations compared to the population Italy. This is in good agreement with previous analyses of other data of some of the samples discussed here (Zimmermann et al., 2017; Diehn et al., 2018).

Comparison of the results for the block scores (**Table 2**, second to sixth section) will help to identify those data blocks that are responsible for a separation based on the global scores. Based on the d-values, a separation of the samples into four groups—corresponding to four environmental conditions—is observed when all populations are analyzed together (last line in each of the sections of **Table 2**). The separation into four groups is possible for each of the five block scores except those of the MALDI block (last line in section 5 of **Table 2**). By interpreting the corresponding dendrogram shown in **Figure S7**, these three groups correspond to the condition 14°C without additional nutrients, 20°C without additional nutrients, and plants that obtained additional nutrients (regardless of growth temperature). The Raman block scores indicate separation of the four groups in the two populations Norway and Italy (**Table 2**, third section). For the other block scores (FTIR, SERS, and MALDI), the separate analysis of each of the populations gives very different results, with the samples from the population Italy showing separation according to the four growth conditions (**Figure 1**) in most of them, but less than four distinct groups in the populations Sweden and Norway. The block scores for the data gathered from the parent plants show very similar behavior and result in clear classification of all four conditions only in population Italy (**Table 2**, sixth section).

**Figure 8:**
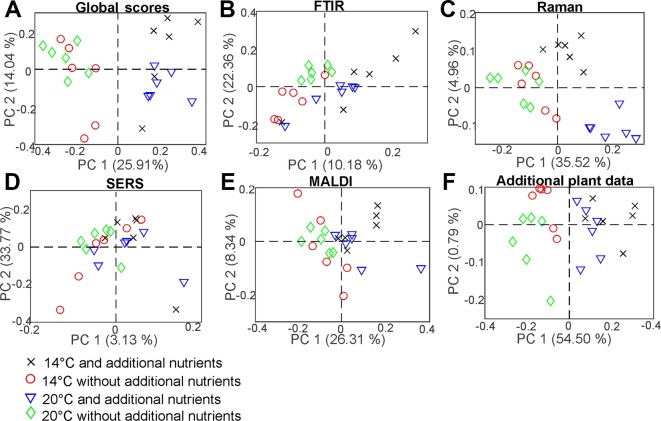
Scores of the CPCA analysis for the classification of samples from pollen of the population Italy regarding the four different growth conditions 14°C and additional nutrients (black crosses), 14°C without additional nutrients (red circles), 20°C and additional nutrients (blue triangles), and 20°C without additional nutrients (green diamonds) for ** (A)** the global scores and ** (B–F) ** the five block scores.

The weighting of each block in the CPCA can be interpreted and allows more insight into the influence the data blocks on each other. As an example, **Figure 8** shows the results for the CPCA applied to the data of the pollen samples from the population Italy. The first component of the global scores plot (**Figure 8A**) separates between positive scores values of the data of samples from progenitor plants that were grown with the addition of nutrients (black crosses and blue triangles) and negative score values for samples from plants that were grown without the addition of nutrients (red circles and green diamonds). In **Figures 8C, F**, the great influence of the Raman and the additional plant data block, respectively, are revealed. Both blocks display similar group formation in the scores plots, with high variances explained by the first CPC of 35.52% and 54.50%, respectively. The corresponding p-values in **Table 2** are very low.

**Figure 9:**
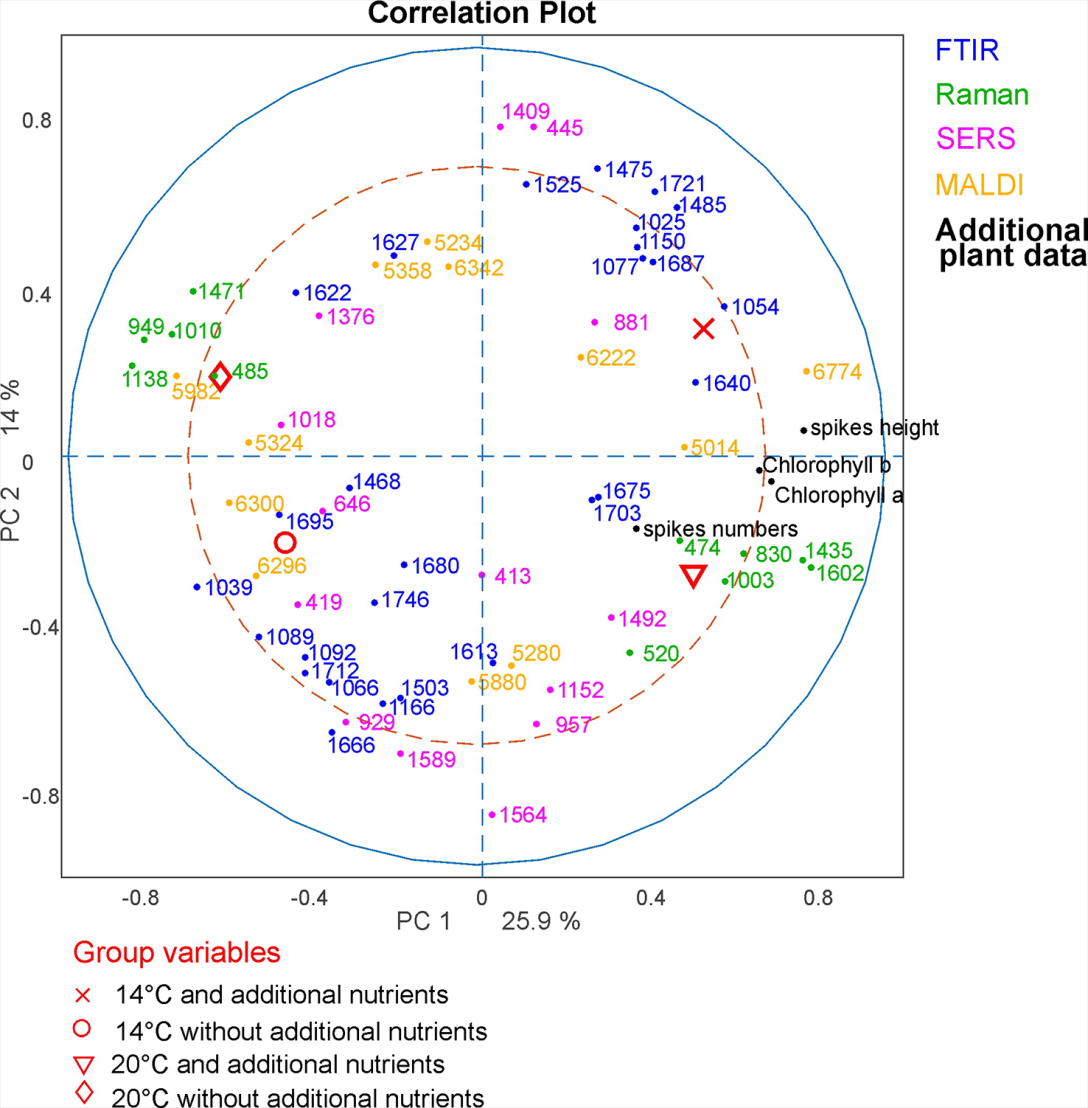
CPCA Correlation loadings plot for the 1st and 2nd CPCA component. Displayed are the global scores of population Italy regarding the four growth conditions 14°C and additional nutrients, (black crosses); 14°C without additional nutrients, (red circles); 20°C and additional nutrients (blue triangles); 20°C without additional nutrients (green diamonds), as well as the loadings of the blocks of FTIR, Raman SERS, MALDI-TOF and additional plant data. For clarity only extrema of the loadings are shown for the spectroscopic/spectrometric data. CPCA, consensus principal component analysis; FTIR, Fourier-transform infrared spectroscopy; Raman, Raman spectroscopy; SERS, surface enhanced Raman scattering; MALDI-TOF MS, matrix assisted laser desorption/ionization mass spectrometry.

The scores of the second CPCA component separate pollen samples grown at 14 °C, as well as at 20 °C without additional nutrients (black crosses, red circles and green diamonds) with positive values from negative values of those pollen samples grown at 20°C without additional nutrients (blue triangles) (**Figure 8A**). The CPC2 is mainly influenced by the SERS data (**Figure 8D**), explaining 33.77% of the variance. In the plot of the block score values (**Figure 8D**), no separation of the groups that could correspond to growth conditions of the plants can be found. This suggests that other sources of variance, in this experiment resulting from individual genotypes, superimpose the influence of the growth conditions as discussed for other data previously (Diehn et al., 2018). It is also in agreement with the calculated p- and d-values for the SERS block (**Table 2**, section 4). Furthermore, the Raman block scores plot, as well as the scores from the additional plant data, show great potential regarding the discrimination of different growth conditions in the population Italy. Since the additional plant data block explains most of the variance in the first CPC, CPCA was also performed without it, by using only the spectroscopic/spectrometric data blocks, in order to confirm that the obtained global pattern is also driven only by the pollen chemical composition (compare with **Figure S8**), not by phenotypic features of the parent plant. Nevertheless, the additional plant data lead to a more complete view in this study and show correlation to the spectroscopic data blocks (compare **Figure 9**).

The molecular differences that cause the separation of the data reveal themselves in the correlation loadings plot for the data from population Italy (**Figure 9**). Again, only those loadings with the highest impact are shown for clarity and only the variables of the additional plant data were presented in full. As expected after the discussion of the block scores (**Figure 8**), the first CPCA component that separates samples from plants grown with additional nutrients (crosses and triangles) from samples without additional nutrients (circles and diamonds, also compare **Figure 8**) is mainly influenced by the Raman block and the additional plant data.

Raman bands that represent pollen samples with nutrient addition are 474, 830, 1003, 1435, and 1602 cm^-1^. The bands at 1435 cm^-1^ and 1602 cm^-1^ can be assigned to lipids (Ivleva et al., 2005; Schulte et al., 2008) and a high mitochondrial activity, respectively (Huang et al., 2004; Pully and Otto, 2009). The other bands are associated with proteins (Schulte et al., 2008; Bagcioglu et al., 2017). The negative scores of the first CPC and the data of the pollen samples without additional nutrients (**Figure 9**, diamonds and circles) are mainly influenced by Raman bands at 485, 949, 1010, 1138, and 1471 cm^-1^. These bands are associated with carbohydrates, such as starch (Schulte et al., 2008; Pigorsch, 2009; Bagcioglu et al., 2017). Pollen are storing their nutrients in lipid bodies as well as in starch bodies, which are occupying most of the space in pollen grains (Wang et al., 2015). Our results confirm that plants growing under different nutrient conditions vary in their quality and/ or amount of such storage bodies inside the pollen.

The second component can be used to separate between rather positive scores values corresponding to samples that were grown at low temperatures (crosses and circles) and negative scores values corresponding to samples that were grown at higher temperatures (diamonds and triangles). As discussed before (compare **Figure 8**), this separation is mainly influenced by SERS and FTIR bands. In particular, samples from plants grown at lower temperatures are characterized by a set of SERS bands that include 445 cm^-1^ and the FTIR bands at 1721 and 1475 cm^-1^. The FTIR signals can be assigned to lipids (Bagcioglu et al., 2015). Samples grown under higher temperatures are characterized by SERS bands at 419, 929, 957, and 1,564 cm^-1^, and FTIR bands at 1,666 and 1,503 cm^-1^. The bands could be assigned to nucleobases and proteins (Seifert et al., 2016). Based on the influence the combination of SERS and FTIR data, we can assume that the discrimination regarding the different growth conditions is probably mostly influenced by the chemical composition of the pollen interior, although –in the preparations for SERS experiments- also water soluble compounds from the pollen outer shell may be found in the aqueous extract.

To summarize the results from the correlations loadings plot, discrimination of different nutrient conditions is mainly influenced by Raman bands that can be assigned to pollen outer shell and nutrient storage, as well as by plant parameters that are present in the additional plant data block. From our results, we infer on differences in amount and quality in lipid and starch bodies inside the pollen grains to be responsible for a distinction of samples from plants grown at different nutrient conditions. This is in good agreement with previous studies on *Poa alpina* using only FTIR spectroscopy (Zimmermann et al., 2017). The temperature conditions at which parent plants are grown mainly affects the SERS and FTIR data blocks and, probably, mainly the chemical composition of the interior of the pollen grains. It has to be pointed out that this conclusion is only made based on the data of the pollen from population Italy, were the samples are showing the highest phenotypic plasticity of the three investigated populations. Within the other populations, the correlation of the signals can differ greatly, indicating higher phenotypic rigidity, as discussed above.

## Conclusion

A well described sample set of pollen from *Poa alpina* was analyzed by FTIR spectroscopy, Raman microspectroscopy, surface enhanced Raman scattering (SERS) and MALDI TOF mass spectrometry, as well as by collected additional data from the parental plants. The chosen methods are complementary regarding sample preparations, selectivity, and sensitivity of the analytical technique. Our results show the ability to detect and describe variances within the pollen composition related either to the place of origin of parent plants (i.e., populations) or the growth conditions. However, suitable data analysis is needed in order to discuss the relatively small chemical differences in these complex biological samples.

The sample set is designed using plants from three populations that were grown under different nutrient conditions and temperatures. Therefore, different levels of classification and influence on pollen composition could be analyzed. As expected, the separation of groups in the sample set according to populations and growth conditions, respectively, is not achieved equally well by each of the methods. As shown by an analysis of different sources of variance using ASCA, different analytical techniques are emphasizing different parameters of pollen chemistry related either to the genetic background or the environmental influence. This has been suggested in previous work where data from FTIR (Zimmermann et al., 2017) and MALDI-TOF-MS (Diehn et al., 2018) on a similar sample set were analyzed but has been shown here using three more, very different types of data. By combination of the different data blocks in a CPCA, a complete set of many differences, observed with the complementary methods can be used to describe the variation with respect to the different groups. We have also compared the individual classification ability of the different methods and the different levels using PCA in combination with simple statistical tests. The different populations can be easily distinguished using MALDI-TOF MS, whereas the three spectroscopic methods are more suitable to separate between different growth conditions. Moreover, as discussed, the same data blocks can have a different influence on the distinction between different growth conditions in the three populations. This implies that, due to the different fraction of the pollen chemistry that is represented by each data block (or analyzed by each of the methods), the biochemical effect of the growth conditions on pollen chemistry can vary for different populations. This would be in agreement with variation in phenotypic plasticity between the populations, in particular regarding different metabolic and molecular pathways used in environmental adaptation.

## Data Availability Statement

The datasets generated for this study are available on request to the corresponding author.

## Author Contributions

AK, BZ, JK, MO, and SF conceived the research idea. SF designed the growth experiments. AK, BZ, and MB designed the FTIR experiments. JK, SD, and SS designed the Raman and SERS experiments. JK, SD, SS, and SW designed the mass spectrometry experiments. BZ and MB performed sampling, FTIR experiments and the measurement of the additional plant data. SD performed the mass spectrometry, Raman and SERS experiments. BZ, SD, and VT analyzed the data. JK and SD wrote the article. AK, BZ, MB, MO, SF, SS, SW, and VT discussed and revised the article.

## Funding

The research was supported by the European Commission through the Seventh Framework Programme (FP7-PEOPLE-2012-IEF project No. 328289) and ERC Grant No.259432 to JK.

## Conflict of Interest

The authors declare that the research was conducted in the absence of any commercial or financial relationships that could be construed as a potential conflict of interest.
