# Supplementary material for: Combining Chemical Information From Grass Pollen in Multimodal Characterization
Source: Front Plant Sci. 2020 Jan 31;10:1788. doi: 10.3389/fpls.2019.01788 (PMC7005252; doi:10.3389/fpls.2019.01788)
Supplement: Supplementary file 1 [file DataSheet_1.pdf]

Supplementary Material

1 Supplementary Figures

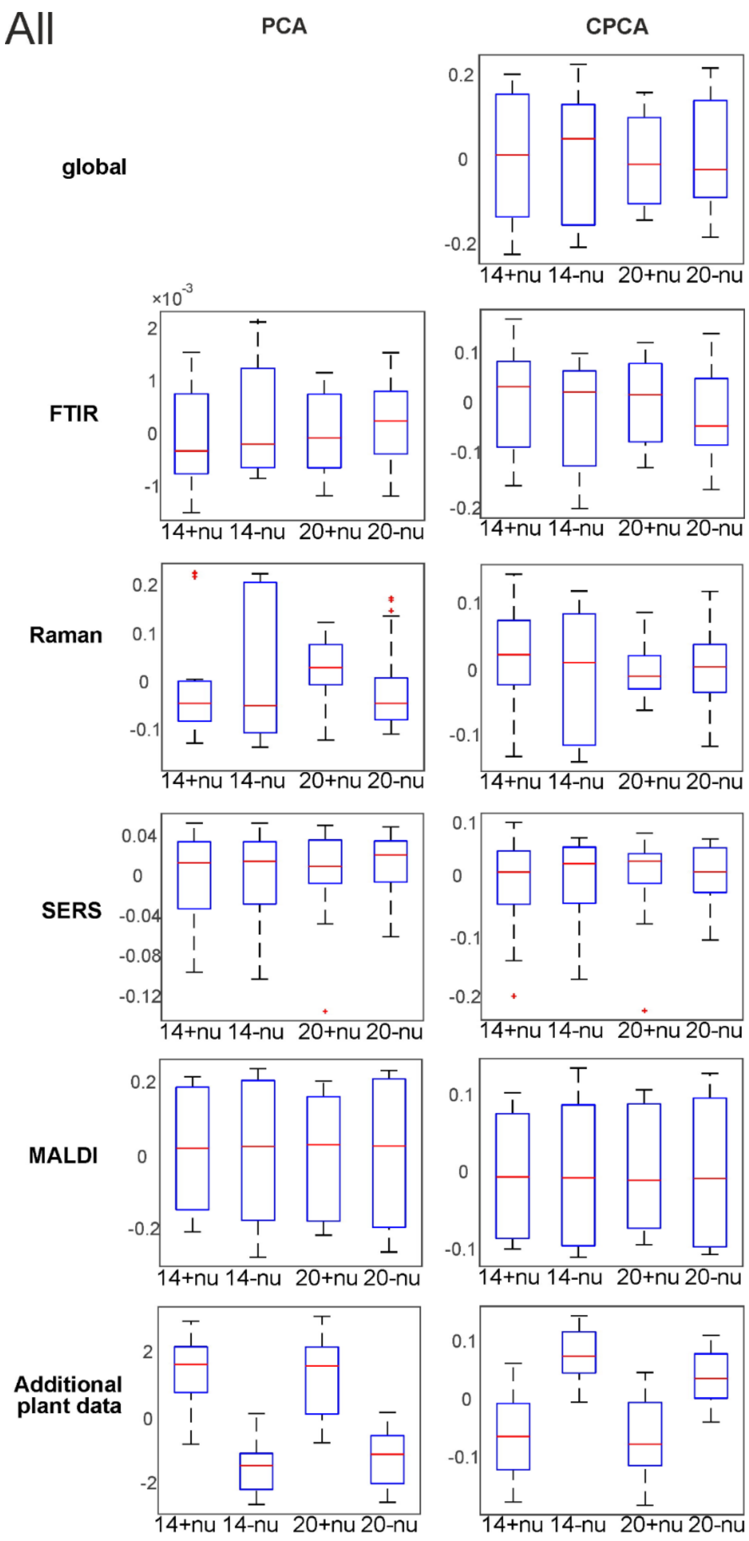

Figure S1 Comparison of the boxplots for the discrimination of the for different growth conditions based on Kruskal-Wallis H-Test of scores from the individual analyses and CPCA. Boxplots for the 5 different methods and boxplots for the global pattern and 5 different blocks after the Multiblock analysis using scores of PC 1 obtained by the whole data set of 72 spectra each.

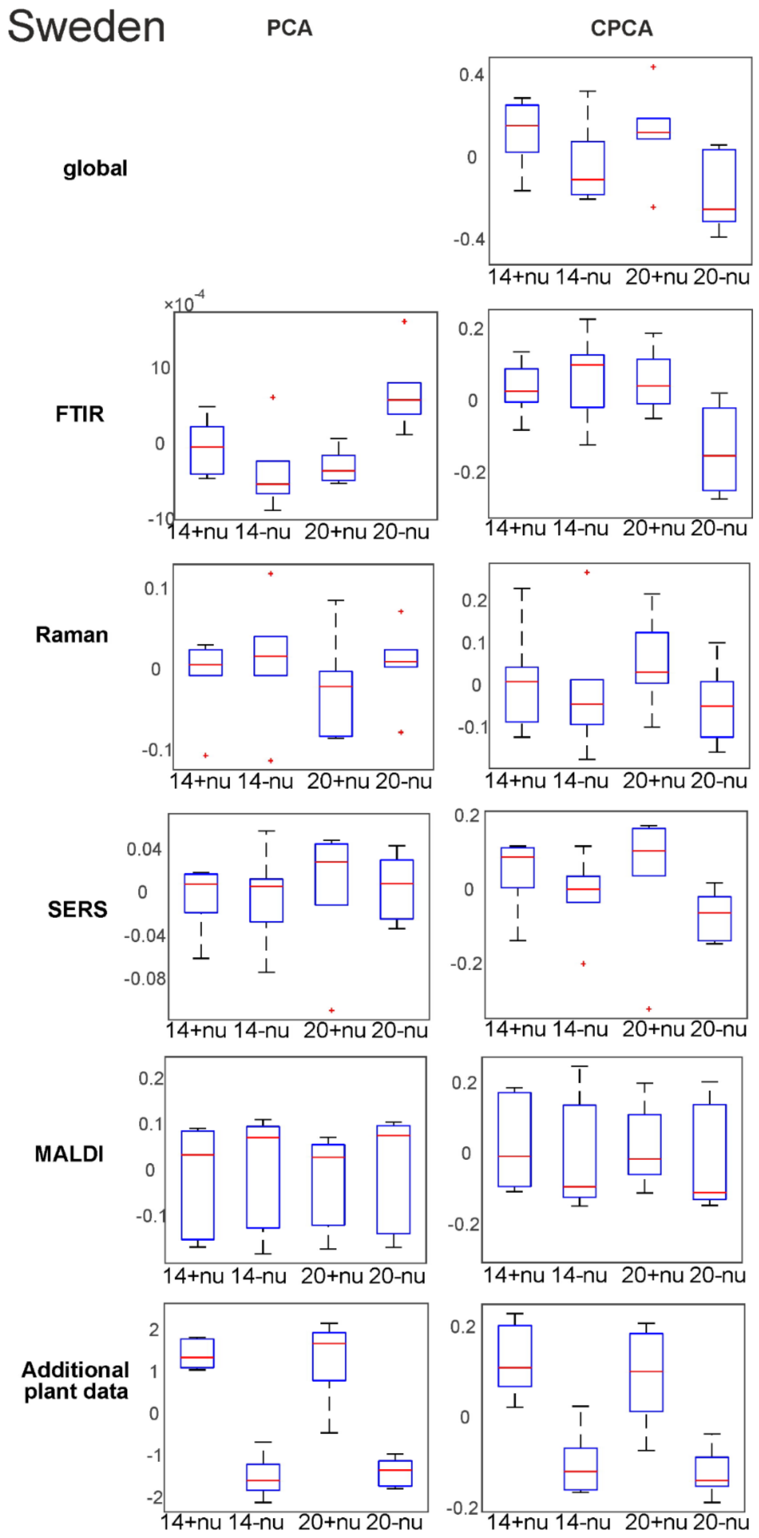

Figure S2 Comparison of the boxplots for the discrimination of the for different growth conditions based on Kruskal-Wallis H-Test of scores from the individual analyses and CPCA. Boxplots for the 5 different methods and boxplots for the global pattern and 5 different blocks after the Multiblock analysis using scores of PC 1 obtained by the data set from the population Sweden of 24 spectra each.

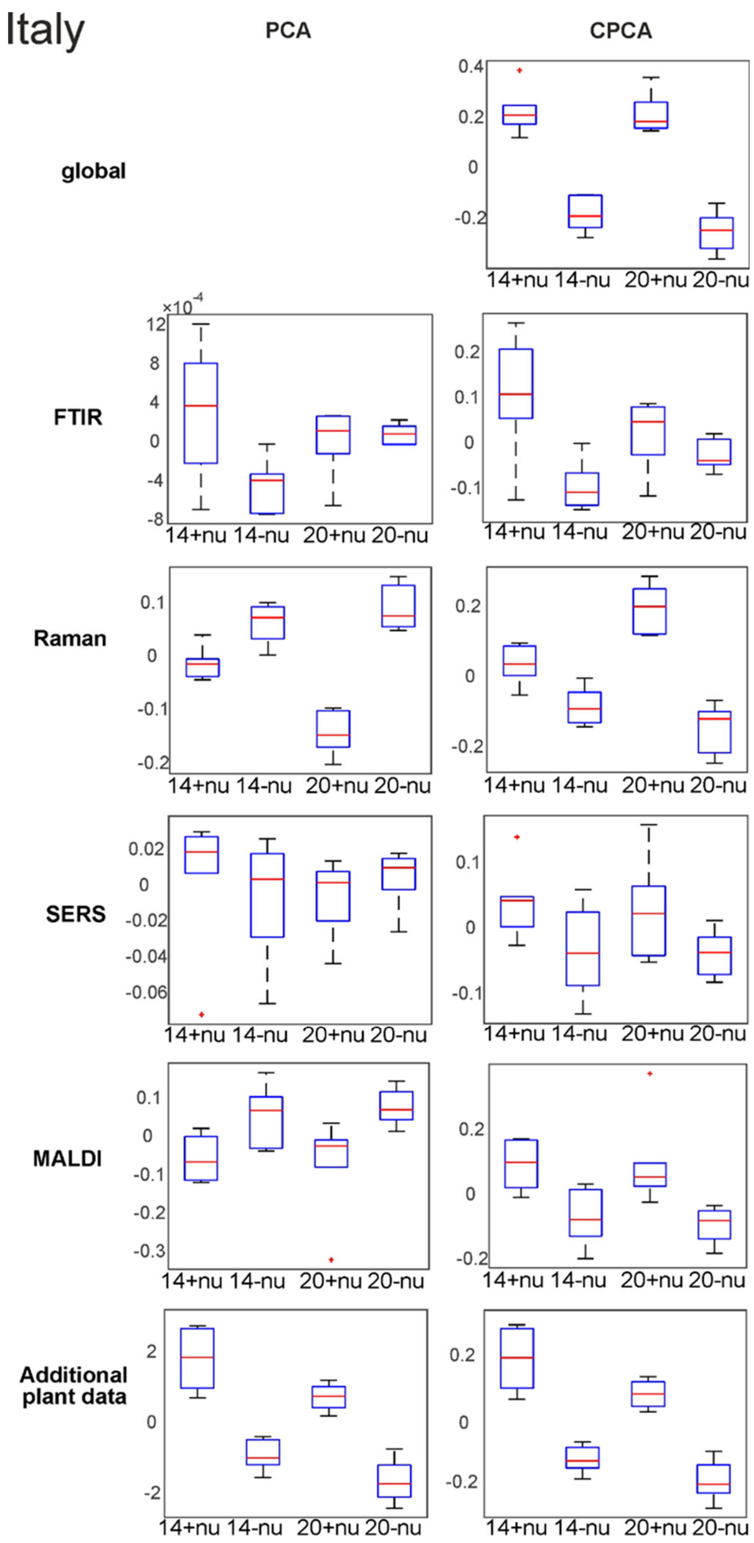

Figure S3 Comparison of the boxplots for the discrimination of the for different growth conditions based on Kruskal-Wallis H-Test of scores from the individual analyses and CPCA. Boxplots for the 5 different methods and boxplots for the global pattern and 5 different blocks after the Multiblock analysis using scores of PC 1 obtained by the data set from the population Italy of 24 spectra each.

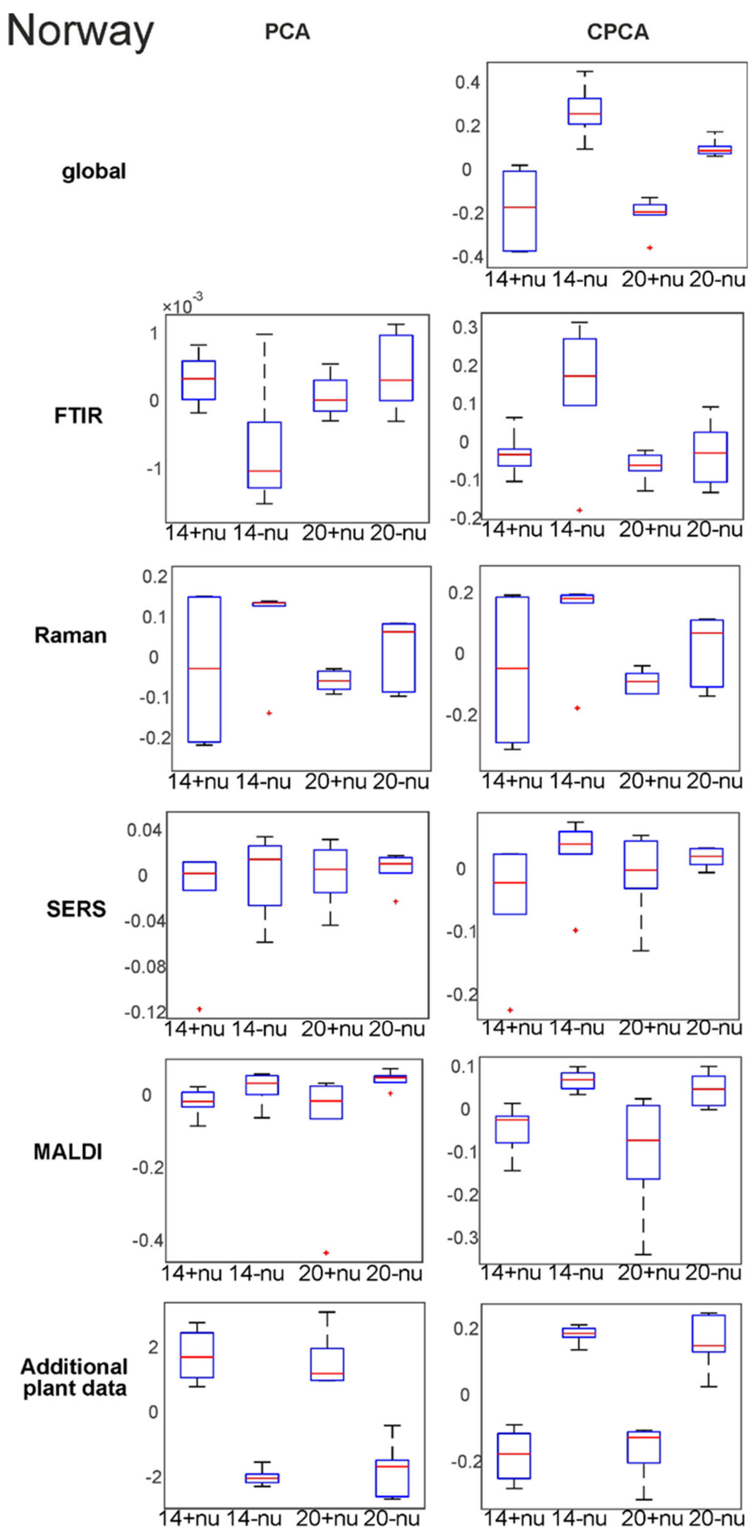

Figure S4 Comparison of the boxplots for the discrimination of the for different growth conditions based on Kruskal-Wallis H-Test of scores from the individual analyses and CPCA. Boxplots for the 5 different methods and boxplots for the global pattern and 5 different blocks after the Multiblock analysis using scores of PC 1 obtained by the data sets of 24 spectra from the population Norway each.

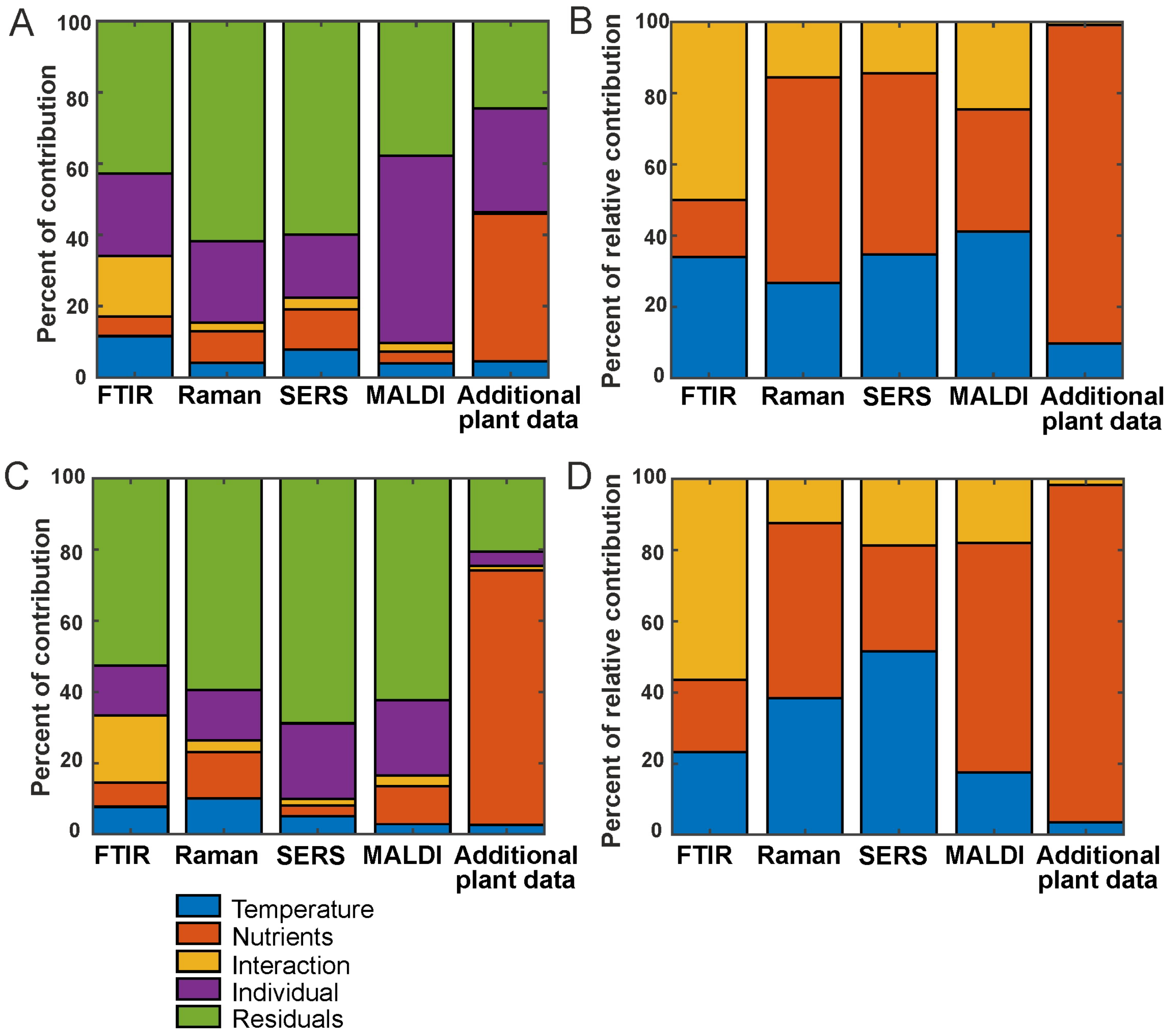

Figure S5 Variation contribution of the different design factors temperature (blue), nutrients (orange), the interaction of temperature and nutrients (yellow), individuals (purple) and the residuals (green) for the 24 spectra from the population Sweden (A and B) and for the 24 spectra from the population Norway (C and D). To emphasize the variations due to growth conditions, the individual variation and residual variation were omitted (B and D).

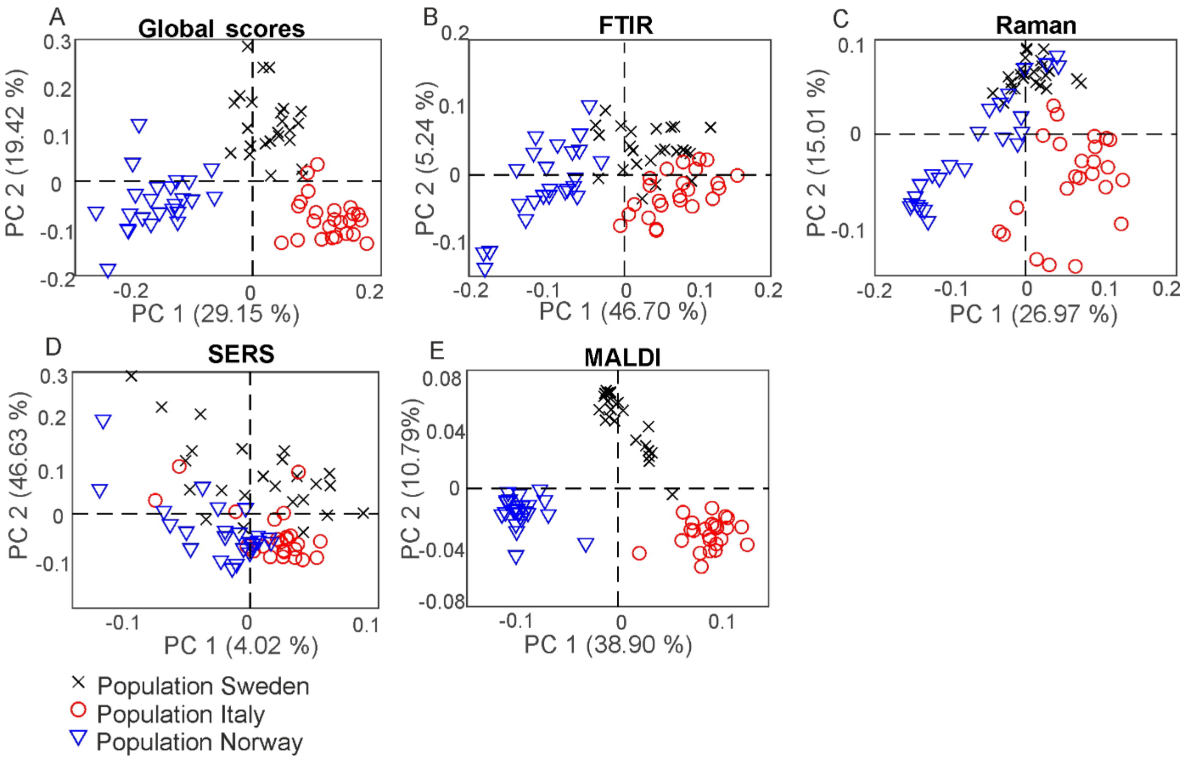

Figure S6 Scores of the CPCA analysis for the classification of samples from the grass pollen populations from Sweden (black crosses), Italy (red circles), and Norway (blue triangles). Shown are the scores for the global scores (A) and the individual data blocks (B-E) and

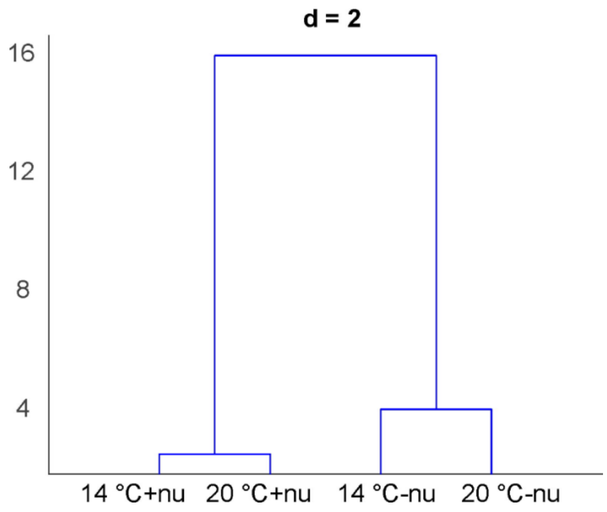

Figure S7 Dendrogram for the MALDI block scores from CPC1 to CPC10 after performing CPCA on all data regarding the different growth conditions.

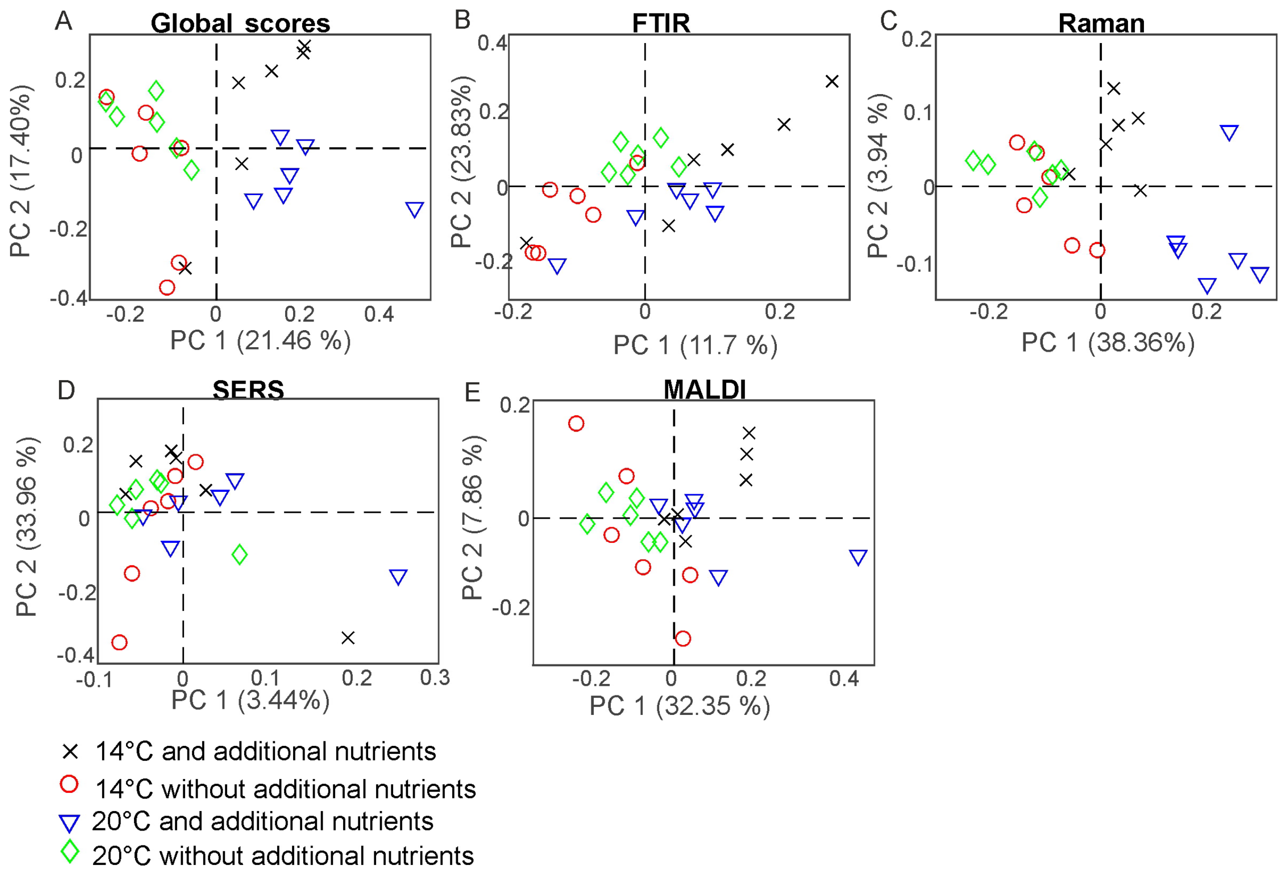

Figure S8 Scores of the CPCA analysis for the classification of samples from the grass pollen the population Italy regarding the four different growth conditions 14 °C and additional nutrients, (black crosses); 14 °C without additional nutrients, (red circles); 20 °C and additional nutrients (blue triangles); 20 °C without additional nutrients (green diamonds).
